# Design and Characterization of Naphthalene Ionic Liquids

**DOI:** 10.3389/fchem.2020.00208

**Published:** 2020-03-24

**Authors:** Verónica Fernández-Stefanuto, Alba Somoza, Raquel Corchero, Emilia Tojo, Ana Soto

**Affiliations:** ^1^Department of Organic Chemistry, Faculty of Chemistry, Universidade de Vigo, Vigo, Spain; ^2^Department of Chemical Engineering, Cretus Institute, Universidade de Santiago de Compostela, Santiago de Compostela, Spain

**Keywords:** synthesis, characterization, surfactant, ionic liquid, enhanced oil recovery

## Abstract

Surfactants have a great number of applications. Among these chemicals, petroleum sulfonates have been widely used due to their effectiveness in reducing interfacial tension. This is the case of sodium octylnaphthalene sulfonate which is a solid with a very low solubility in water. To overcome these drawbacks, this work aimed to synthesize new surface active ionic liquids based on a naphthalene sulfonate anion and traditional cations of these salts (imidazolium, pyrrolidinium, and pyridinium). The new chemicals showed high thermal stability, ionic liquid nature, and a stronger surfactant character than the original naphthalene. Moreover, they were found to be water soluble which greatly facilitates their application in the form of aqueous formulations. 1-Hexyl-3-methylimidazolium 4-(*n*-octyl)naphthalene-1-sulfonate showed the best capacity to reduce water-air and water-oil interfacial tension.

## Introduction

Surfactants are extensively used not only in the chemical industry but also in daily life. Applications include: detergents, emulsifiers, de-emulsifiers, dispersants, lubricant additives, wetting agents, corrosion inhibitors, foaming agents, enhancing additives in oil recovery, phase transfer or drug delivery agents, chemical reaction media (e.g., for micellar catalysis), etc. Most applications of surface active agents derive from two fundamental properties in aqueous solution: adsorption at the interface and aggregation. Adsorption at the air/water interface allows reduction of the surface tension and modification of wetting and foam-forming properties of the surfactant-containing water. Adsorption at the oil/water interface is the first step in emulsification, while at the interface of water with suspended solids, it affects flocculation and coagulation. The aggregation of surfactants is the basis of applications involving the formation of micelles, microemulsions, and liquid crystals. Thus, the study of the behavior of these chemicals in water is the first step in their application.

Among the surfactants, petroleum sulfonates have been widely used because they are effective at attaining low interfacial tension, relatively inexpensive and chemically stable. Petroleum sulfonates are produced when an intermediate-molecular-weight refinery stream is sulfonated, and synthetic sulfonates are the result of sulfonating a relatively pure organic compound (Green and Willhite, 1998). The first synthetic surfactants based on fossil raw materials were the alkylnaphthalenesulfonates. They are used in many applications as detailed below. They offer acid, base and thermal stability. They have excellent wetting and dispersing properties, and also can be designed to have different foaming tendencies. Abdel-Raouf et al. (2011) synthesized several alkylnaphthalene and alkylphenanthrene sulfonates by means of a Wurtz–Fittig reaction. Aggregation properties were studied in water and the detergency power of the prepared surfactants shown. These kinds of surfactants are also used as lubricant additives because they impart thermal and thermo-oxidative stability. The electron-rich naphthalene ring has the ability to absorb energy, resonate, and then disperse that energy (Hunter, 2017). Finally, perhaps one of the most promising applications for these compounds is Enhanced Oil Recovery (EOR). Their ability to reduce water/oil interfacial tension make them desired components in surfactant or microemulsion formulations designed to liberate oil trapped in the pores of rocks.

Gong et al. (2005) synthesized sodium and sodium methyl naphthalene sulfonates by naphthalene sulfonation with chlorosulfonic acid in carbon tetrachloride at low temperature, followed by neutralization with NaOH solution. The interfacial tension (IFT) between these surfactants and Liaohe or Shengli crude oil was measured. It was found that the rigid aromatic ring could play the role of a hydrophobic group and that the effect of the alkyl group was not very significant. The surfactants proved effective in lowering the IFT. However, formulations with NaCl, alkali, and other surfactants were required to obtain ultra-low interfacial tension (~10^−3^ mN/m). Wurtz–Fittig and sulfonation reactions were also used by Tan et al. (2004a) to synthesize sodium alkylnaphthalene sulfonates with longer alkyl chains (C_6_, C_8_, C_10_). Comparing the aggregation capacity of different sulfonate surfactants, they found that critical micelle concentration decreases according to *n*-alkyl > *n*-alkylbenzene > *n-*alkylnaphthalene. Moreover, in the case of the latter, the higher the alkyl chain length, the lower the critical micelle concentration (cmc) of these surfactants in water. Similar conclusions were obtained by these authors (Tan et al., 2004b) using fluorescence techniques instead of surface tension measurements to characterize the micellization and microenvironmental properties of these surfactants. Optimal formulations for EOR containing sodium alkylnaphtalene sulfonates were defined by Chu et al. (2004). Ultra-low interfacial tensions were achieved in surfactant/alkali/acidic oil systems. The synergism between the sulfonates and surface-active components in crude oil was controlled by sulfonate and alkali concentration, alkaline type and ionic strength. The alkylation of β-methylnaphthalene with a series of different chain lengths of *n*-bromoalkanes led to the synthesis of alkyl (C_6_-C_14_) methylnaphthalene sulfonate surfactants. All of the synthesized surfactants except for hexyl methylnaphthalene sulfonate were able to reduce the interfacial tension between Shengli oil and water to ultra-low values in the absence of alkali (Zhao et al., 2006, 2007).

The design of new sulfonate surfactants based on the naphthalene ring was accomplished by Berger and Lee (2002). They carried out the simultaneous sulfonation and alkylation of aromatic compounds using olefin sulfonic acids. Alkylnaphthalene sulfonic acids were obtained, among other surfactants, but in this case the sulfonic group was attached to the end of the alkyl chain instead of the aromatic ring as usual. The authors propose these new compounds not only for use in EOR but also as dispersants and emulsifiers for cleaners, detergents, agriculture, oilfield drilling muds, cement, metal treating, etc.

One of the most highlighted properties of ionic liquids (ILs) is their tunability. By selecting the ions, the alkyl chains, functional groups, etc. a great number of chemical designs which improve the characteristics or the applicability of these chemicals are possible (Pirkwieser et al., 2018; Lethesh et al., 2019). With this idea in mind, and due to the interest in alkylnaphtalene sulfonate surfactants, the aim of this work is the design of new kinds of surfactants containing this anion and typical IL cations (imidazolium, pyrrolidinium and pyridinium). An imidazolium ring was considered as the archetypal cation of ILs, and pyrrolidinium and pyridinium as more biodegradable alternatives. An alkyl chain length able to produce ILs with high solubility in water and low cmc was selected. The objectives include reducing the melting point of these traditional surfactants, and analyzing the aggregation capacity of the resultant chemicals.

## Materials and Methods

Reagents used in the metathesis reactions were prepared as reported in literature: N-methylpyrrolidinium dihydrogen phosphate [C_1_Pyr][H_2_PO_4_] (Pal and Saini, 2019), 1-hexyl-3-methylimidazolium chloride [C_1_C_6_Im]Cl (Min et al., 2006), 1-ethylpyridinium bromide [C_2_Py]Br (Potangale et al., 2017), and 1-butylpyridinium chloride [C_4_Py]Cl (Potangale et al., 2017). These starting materials were purified as indicated in Supplementary Material. That is: Na[ONS] (2) was recrystallized from an ethanol/water mixture (50:50, vol/vol), [C_1_C_6_Im]Cl, [C_2_Py]Br, and [C_4_Py]Cl were washed with AcOEt to remove possible unreacted starting reagents and dried under reduced pressure, while [C_1_Pyr][H_2_PO_4_] was just heated under reduced pressure to remove volatile starting materials. From the NMR spectra and MS data a purity ≥99%wt was estimated for all of them. Tetrahydrofuran was distilled from sodium/benzophenone prior to use. All other materials were reagent grade purchased from commercial suppliers: Acros Organics (*n*-octylmagnesium bromide 2M solution in diethyl ether, zinc chloride > 97%wt, 1-bromonaphtalene 96%wt, chlorosulfonic acid 97%wt) and Sigma Aldrich (Ni(dppp)Cl_2_ ≤ 100%wt), and employed without further purification.

The chemicals used in the dynamic interfacial tension measurements were: *n*-octane purchased from Sigma-Aldrich with a purity >99%wt, and crude oil kindly supplied by Repsol (A Coruña, Spain). Main properties of the oil are shown in Table 1.

**Table 1:** Crude oil properties (provided by supplier).

Density at 288.15 K (kg/m^3^)	811.1
Reid vapor pressure (kPa)	44.9
Viscosity at 293.15 K (cSt)	4.861
Carbon residue (%wt)	1.2522
Asphaltenes (%wt)	0.4624


The glass material employed in the synthetic reactions was dried in an oven at 333 K for 24 h before use. The evolution of the reactions was monitored by thin layer chromatography (t.l.c.) employing silica-gel sheets (Merck, TLC Silica gel 60 F254). Spectroscopic data were provided by the Center of Scientific-Technological Support to Research (CACTI) of the University of Vigo. ^1^H and ^13^C NMR spectra were recorded on a BRUKER ARX 4CO spectrometer at 400.1621 (^1^H) and 100.6314 (^13^C) MHz, respectively. CDCl_3_ (ACROS Organics, 99.6+ atom % D) and D_2_O (ACROS Organics, 99.8+ atom % D) were employed as deuterated solvents. Chemical shifts are quoted in parts per million (ppm) relative to the signals corresponding to the residual non-deuterated solvents (CDCl_3_: δH = 7.26 ppm, δC = 77.16 ppm). Coupling constants are given in hertz (Hz). Low and high resolution ESI mass spectra were recorded on a BRUKER FTMS APEXIII spectrometer, while ICP-MS analyses were carried out on an X Series ICP-MS (Thermo Elemental) equipment. All solutions for ICP-MS analyses were prepared using ultra-high purity Milli-Q water.

Karl-Fischer titration was used to measure water content of the original surfactant and synthesized ILs. In the case of the solid materials, they were solubilized in 2-propanol and their water content corrected according to the water content of the solvent.

Thermogravimetric analysis (TGA) and differential scanning calorimetry (DSC) were used to characterize the synthesized ILs. Thermal decomposition of the samples was analyzed in a TA Instruments Q500 thermogravimetric analyzer with a weight precision of ±0.01%. This apparatus was weight-calibrated with weights of certified mass, and temperature-calibrated with nickel of high purity (99.9945%wt) by means of the determination of its Curie temperature. An open platinum pan loaded with ~12 mg of sample was used in each case. A simple heating ramp of 10 K·min^−1^ from room temperature to 900 K was applied, using nitrogen gas (Praxair, 99.999%) as both balance purge gas (40 mL·min^−1^) and sample purge gas (60 mL·min^−1^). In addition, since it is well-known that isothermal experiments give safer values of temperature operation for ILs, isothermal studies were also carried out at different temperatures. All the experiments were performed at least twice to ensure repeatability.

DSC runs were carried out in a TA Instruments Q2000 differential scanning calorimeter, equipped with an RCS 90 refrigerated cooling system. The apparatus was calibrated with high purity indium (99.99%wt) by means of the determination of its onset melting temperature. Approximately 12 mg of each sample were placed in a 40 μL aluminum pan, sealed hermetically with a lid of the same material and loaded into the measuring chamber with an autosampler. An identical empty pan with its corresponding lid was used as reference. Nitrogen gas (Praxair, 99.999%wt), at a flowrate of 50 mL·min^−1^, was used as sample purge gas. The thermal program consisted of a cooling ramp and then a heating ramp from 190 K to a temperature below the decomposition at a rate of 5 K·min^−1^. The procedure was repeated, with intercalated 10-min isotherms at both ends of the temperature range, and when the curves were coincident (usually in the second and third cycle) results were analyzed. A second study was carried out at 3 K·min^−1^ in order to confirm results obtained at the highest rate.

Surface tensions of the synthesized ILs in aqueous solutions were measured using the Wilhelmy plate method in a Krüss K11 tensiometer in order to determine the cmc. An external thermostat (Selecta Frigiterm 6000382) maintained the temperature at 298.15 K. Dynamic interfacial tensions between the surfactant IL solutions and octane or crude oil were measured using a Krüss SITE 100 spinning drop tensiometer. Temperature was controlled circulating oil from a thermostatic bath Julabo model EH-5. The capillary was filled with the heavy phase (aqueous solution) and a drop of the light phase (oil) was injected. Rotating velocities between 3000 and 7000 rpm were applied. The interfacial tension was calculated according to the following equation:

(1) $$\begin{array}{r} \gamma =\frac{\Delta \rho \omega^{2}D^{3}}{32} \end{array}$$

where ω is the angular velocity, D is the diameter of the oil drop and Δρ the density difference between the aqueous phase and the oil. All the experiments were performed at least twice to ensure repeatability.

## Results and Discussion

### Synthesis

**Scheme 1:**
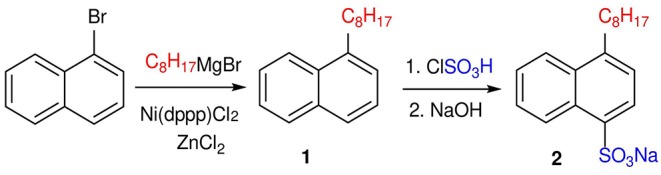
Synthetic procedure applied to obtain sodium 4-octylnaphthalene-1-sulfonate (2).

**Scheme 2:**
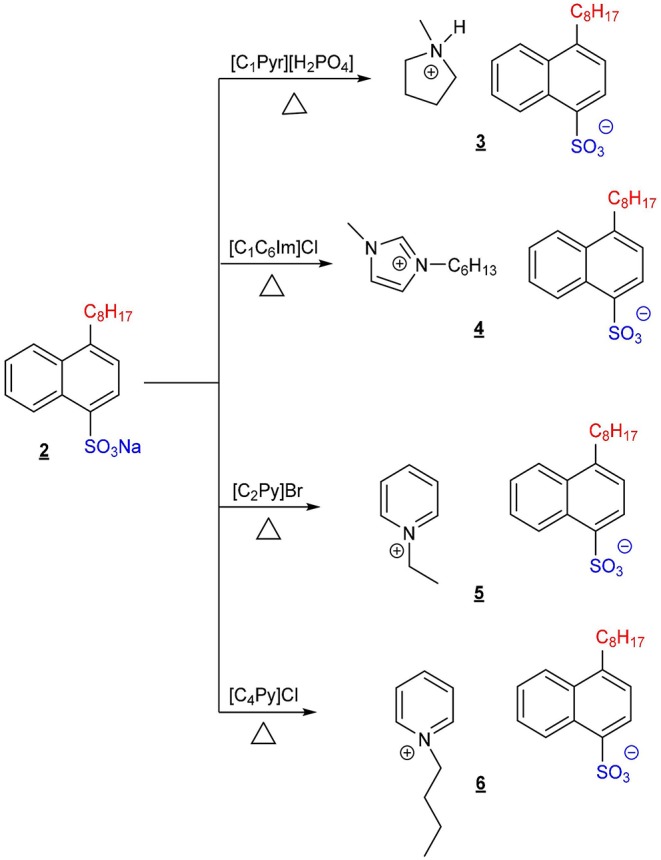
General synthetic procedure for the methatesis reactions applied to obtain the ILs described in this work.

The ILs described in this work were obtained by applying a synthetic procedure of three steps (Schemes 1, 2). The first one involved the alkylation of 1-bromonaphthalene in the presence of Ni(dppp)Cl_2_ and ZnCl_2_ to obtain 1-*n*-octylnaphthalene (1) with high yield (Palmaerts et al., 2009; Nietfeld et al., 2011). The later sulfonation of 1-*n*-octylnaphthalene (1) by treatment with chlorosulfonic acid and neutralization with NaOH gave sodium 4-(*n*-octyl)naphthalene-1-sulfonate Na[ONS] (2) with a high yield as the only product after recrystallization (Tan et al., 2004a). Finally, the exchange of Na^+^ for the desired cations was carried out by the corresponding metathesis reactions of sulfonate 2 with the ILs N-methylpyrrolidinium dihydrogen phosphate [C_1_Pyr][H_2_PO_4_], 1-hexyl-3-methylimidazolium chloride [C_l_C_6_Im]Cl, 1-ethylpyridinium bromide [C_2_Py]Br, and 1-butylpyridinium chloride [C_4_Py]Cl.

The resulting reaction products of the metathesis reactions were dissolved in CH_2_Cl_2_ to precipitate the formed inorganic salt (NaH_2_PO_4_, NaCl or NaBr), which was filtered off. The filtrate was then concentrated and dried under high vacuum (2 × 10^−1^ Pa) to obtain alkylnaphthalene-ILs: N-methylpirrolidinium 4-(*n*-octyl)naphthalene-1-sulfonate [C_1_Pyr][ONS] (3), 1-hexyl-3-methylimidazolium 4-(*n*-octyl)naphthalene-1-sulfonate [C_1_C_6_Im][ONS] (4), 1-ethylpyridinium 4-(*n*-octyl)naphthalene-1-sulfonate [C_2_Py][ONS] (5) and 1-butylpyridinium 4-(*n*-octyl)naphthalene-1-sulfonate [C_4_Py][ONS] (6). The ^1^H and ^13^C NMR spectra of the synthesized ILs, as well as their HR-MS data, confirmed their structure and showed the absence of unreacted starting materials. The base peak observed in the HR-MS spectra corresponded to [A_2_B]^+^ ion associations (A = cation mass, B = anion mass), frequently observed in ILs MS spectra. In addition, the ICP-MS analyses allowed quantifying the concentration of the inorganic ions that could remain after the metathesis reactions. It was found to be lower than 0.17%wt in all cases (see Supplementary Material). From the NMR spectra and MS data, a purity ≥99%wt is estimated for all the ILs synthesized in this study. Water content of ILs is also presented in Supplementary Material.

All of the synthesized ILs were found to be water soluble.

### Thermal Characterization

**Figure 1:**
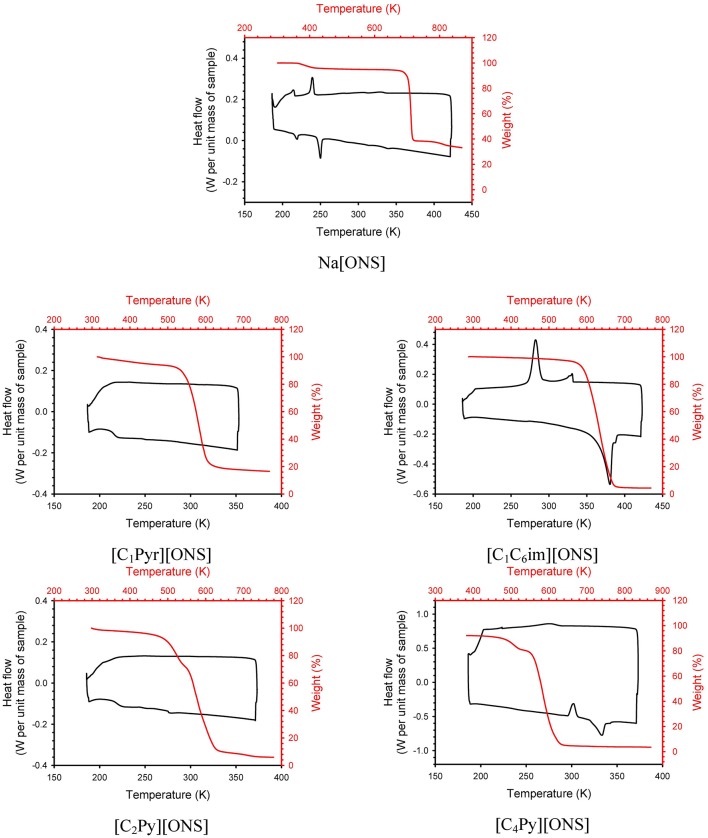
DSC and TGA curves of surfactants.

The thermal stability of the original surfactant (sodium 4-(n-octyl)naphthalene-1-sulfonate) and the four synthesized surfactant ILs was investigated via TGA experiments. TGA curves can be seen in Figure 1. Table 2 shows the 5% onset decomposition temperature (T_d, 5%onset_), a more conservative value than the usual T_d, onset_ in order to define a safe temperature of operation with these ILs. As could be expected, the incorporation of a large and asymmetrical organic cation leads to a decrease of the thermal stability. Isothermal scans at temperatures lower than calculated T_d, 5%onset_ are presented in Supplementary Material. The influence of the cation on the thermal stability is confirmed, with a decrease of the stability according to: Na > [C_1_C_6_Im] > [C_1_Pyr] > [C_4_Py] > [C_2_Py]. However, at least 100 K below T_d, 5%onset_ are required in order to have insignificant loss weigh when surfactants are maintained at high temperature for a long period of time.

**Table 2:** Thermal characterization of surfactants at 0.1 MPa.

**Surfactant**	***T_***c***_(K)***	***T_***cc***_(K)***	***T_***ss*−*Cool***_(K)***	***T_***g***_(K)***	***T_***ss*−*Heat***_(K)***	***T_***m***_(K)***	***T_***d, 5*%*onset***_ (K)***
Na[ONS]			215/236		215/246		686
[C_1_Pyr][ONS]				216			522
[C_1_C_6_Im][ONS]	289/332					367	571
[C_2_Py][ONS]				219			473
[C_4_Py][ONS]		296				323	488

*Standard uncertainties: u(P) = 5 kPa, u(T) = 1 K*.

Thermal events for the ILs were investigated via DSC experiments and results at a working rate of 5 K/min are shown in Table 2 (Figure 1 shows the third cycle). Temperatures were calculated as the extrapolated onset-temperature. This temperature is the intersection point of the extrapolated baseline and the inflectional tangent at the beginning of the peak. The baseline and the inflectional tangent are determined from the temperature-dependent heat flow signal. In contrast to peak-temperature, the onset-temperature is less dependent on heating rate and sample mass. In the case of the Na[ONS] surfactant, two endothermic peaks were found in the heating ramp and two exothermic peaks in the cooling ramp, at 215/236 and 215/246 K, respectively. No other peaks were found heating up to the decomposition temperature of this chemical. In order to confirm that these thermal events correspond to solid-solid transitions, a sample was heated in an oven up to 473 K and no melting was observed. [C_1_Pyr][ONS] has a glass transition temperature at 216 K. It is a viscous liquid at room temperature. [C_1_C_6_Im][ONS] shows melting at 367 K and crystallization at 332K. Another small exothermic peak appears in the cooling ramp at 289 K. This suggests a polymorphic-like behavior that leads to the formation of crystals with different structures (Calvar et al., 2013; Villanueva et al., 2015). In the case of the surfactants with a pyridinium cation, their thermograms are quite different despite the similarity of their structures. Only a glass transition temperature (219 K) was detected in the case of [C_2_Py][ONS], however it has a solid appearance at room temperature. In the case of [C_4_Py][ONS] a different profile was observed. A cold crystallization peak appears at 296 K and a melting point at 323 K. In order to check the nature of the peaks obtained, DSC runs were also carried out at a rate of 3 K/min (Supplementary Material shows all the cycles at this rate). All the thermal events presented in Table 2 are confirmed (kinetic events appearing at higher temperatures). Only in the case of [C_1_C_6_Im][ONS] a new cold crystallization was observed. This is not unusual. The ILs possess great crystallization and glass-forming ability (Lobo Ferreira et al., 2019) strongly dependent on their water content and operation conditions.

### Aggregation in Water

The critical micelle concentration (cmc) of the original surfactant and the synthesized ILs was measured in aqueous solution at 298.15 K using surface tension measurements. Results can be seen in Table 3. In the case of the surfactant Na[ONS], this parameter was previously published. Tan et al. (2004a,b) obtained at 303.15 K a cmc value of 2.36 mmol/L with a surface tension at this concentration (γ_*cmc*_= 39.3 mN/m) while Abdel-Raouf et al. (2011) obtained at 298.15 K a value of 20.7 mmol/L (γ_*cmc*_ = 33.4 mN/m), both using surface tension measurements. Due to the discrepancy of these values, obtained with the same experimental technique, the determination of this parameter at 298.15 K was repeated in this work and a value of 3.86 mmol/kg (γ_*cmc*_ = 41.0 mN/m) was obtained. Results are in agreement with values obtained by Tan et al. (2004a,b).

**Table 3:** cmc and surface tension of surfactants in aqueous solutions at 298.15 K and 0.1 MPa.

**Surfactant**	***cmc* (mmol/kg)**	***γ_*cmc*_* (mN/m)**
Na[ONS]	3.86	41.0
[C_1_Pyr][ONS]	0.78	37.4
[C_1_C_6_Im][ONS]	0.39	29.7
[C_2_Py][ONS]	1.17	36.9
[C_4_Py][ONS]	0.74	36.8

*Standard uncertainties: u(P) = 5 kPa, u(T) = 0.05 K, u(cmc) = 0.01, u(γ) = 0.3 mN/m*.

**Figure 2:**
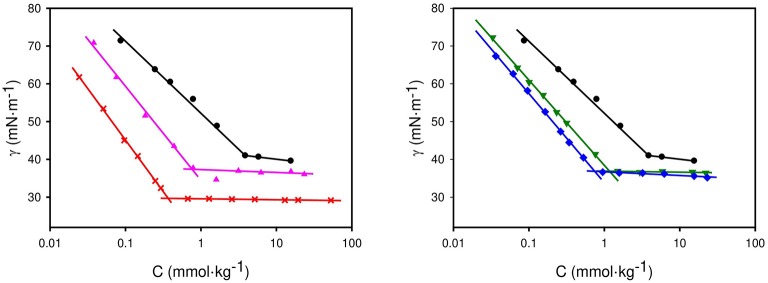
Surface tension of aqueous solutions of surfactants at 298.15 K. • Na [ONS] ,**▴**[C_1_Pyr] [ONS] ,**×**[C_1_C_6_Im] [ONS] ,▾[C_2_Py] [ONS] ,♦[C_4_Py] [ONS].

Regarding the capacity of aggregation of the synthesized ILs, Figure 2 shows the determination of the cmc and results presented in Table 3. In comparison with the original surfactant, all the ILs have lower cmc values (ranging from 0.4 to 1.2 mmol/kg) and higher capacity to reduce the interfacial tension air/water (values ranging from 29.7 to 37.4 mN/m). The IL that led to the lowest cmc, and the greatest reduction of water surface tension was the imidazolium, and in the case of the pyridinium ILs, the lower the alkyl chain length the higher the cmc, with little variation in surface tension.

### Dynamic Interfacial Tension

**Figure 3:**
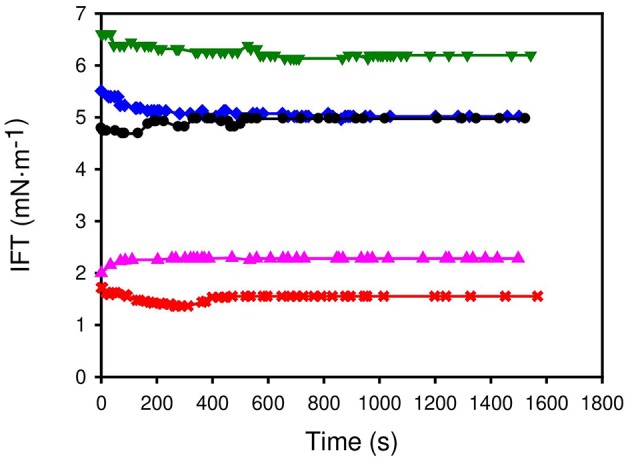
Dynamic interfacial tension between aqueous solutions of the surfactants (concentration is twice the cmc) and *n-*octane. • Na [ONS] ,**▴**[C_1_Pyr] [ONS] ,**×**[C_1_C_6_Im] [ONS] ,▾[C_2_Py] [ONS] ,♦[C_4_Py] [ONS].

Applications of surface active compounds require that these chemicals drastically reduce the surface tension of water and its interfacial tension against oils. Thus, *n*-octane was selected as oil and the dynamic interfacial tension water-octane was measured at 298.15 K using aqueous solutions of the original surfactant and new synthesized ILs. A concentration of surfactant in the aqueous solution equal to twice the value of the cmc was selected for each surfactant. Figure 3 shows the results. [C_1_C_6_Im][ONS] and [C_1_Pyr][ONS] have the capacity of lowering the interfacial tension more than the original surfactant Na[ONS] using lower concentrations. The efficiency in IFT reduction decreases in the following order: [C_1_C_6_Im][ONS] > [C_1_Pyr][ONS] > Na[ONS] ~ [C_4_Py][ONS] > [C_2_Py][ONS]. In this case, the longer alkyl chain of pyridinium ILs led to a better interaction with the oil, thus reducing the IFT.

**Figure 4:**
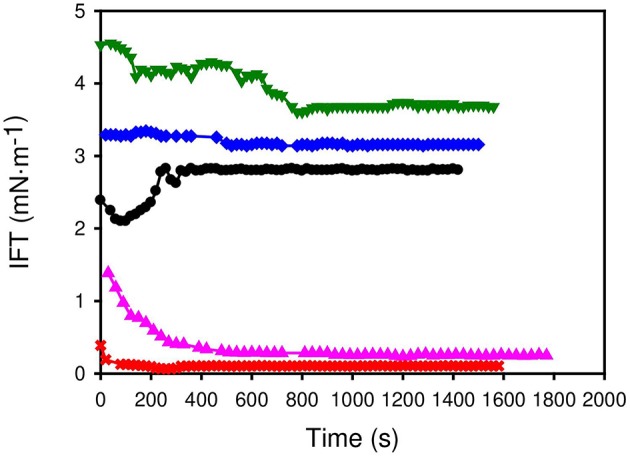
Dynamic interfacial tension between aqueous solutions of the surfactants (concentration is twice the cmc) and crude oil. • Na [ONS] ,**▴**[C_1_Pyr] [ONS] ,**×**[C_1_C_6_Im] [ONS] ,▾[C_2_Py] [ONS] ,♦[C_4_Py] [ONS].

With EOR applications in mind, the water-crude oil interfacial tension was also measured at 298.15 K. Similar results (see Figure 4) to the case of *n-*octane were found. Equilibrium interfacial tensions achieved a range from 3.5 mN/m in the case of [C_2_Py][ONS] to 0.1 mN/m for [C_1_C_6_im][ONS].

## Conclusions

New alkylnaphthalenesulfonate surface active ILs were designed and successfully synthesized with high purity, namely: [C_1_Pyr][ONS], [C_1_C_6_Im][ONS], [C_2_Py][ONS] and [C_4_Py][ONS]. A synthetic procedure of three steps was required. First, sodium 4-(*n*-octyl)naphthalene-1-sulfonate, Na[ONS], was obtained by alkylation of 1-bromonaphthalene followed by sulfonation and later neutralization. Then the desired cations were introduced by the corresponding metathesis reaction with previously synthesized simple ILs. Unlike the starting product, Na[ONS], the new chemicals based on traditional IL cations (imidazolium, pyrrolidinium, and pyridinium) showed high solubility in water, which greatly favors their application in the form of aqueous formulations.

The incorporation of a large organic cation to the 4-(*n*-octyl)naphthalene-1-sulfonate anion led to a decrease in the glass transition/melting point of the synthesized compounds, and their IL nature was confirmed. Moreover, [C_1_Pyr][ONS] is liquid at room temperature and pressure which facilitates its manipulation at these conditions. Even when the inclusion of those cations decreased the decomposition temperatures in comparison to the starting product, the new chemicals can be used in a wide range of temperatures without suffering decomposition.

All the ILs self-aggregate in water, obtaining in all cases lower cmc and higher reduction of the surface tension of water than with Na[ONS]. This indicates that lesser amounts of the chemicals would be required if the ILs were selected for any application. Regarding the water/octane interfacial tension, [C_1_C_6_Im][ONS] and [C_1_Pyr][ONS] are also able to reduce it to a greater extent than Na[ONS] with lower concentration of surfactant. Aiming at EOR applications, a very significant reduction of water-crude oil IFT was found with [C_1_C_6_Im][ONS] (~0.1 mN/m). To achieve ultra-low values, as in the case of other naphthalene sulfonate surfactants (Chu et al., 2004; Gong et al., 2005), these chemicals must be formulated with electrolytes, co-surfactants, etc. to offer a promising formulation for this application.

## Data Availability Statement

The datasets generated for this study are available on request to the corresponding author.

## Author Contributions

All authors listed have made a substantial, direct and intellectual contribution to the work, and approved it for publication.

### Conflict of Interest

The authors declare that the research was conducted in the absence of any commercial or financial relationships that could be construed as a potential conflict of interest.
