# Supplementary material for: Design and Characterization of Naphthalene Ionic Liquids
Source: Front Chem. 2020 Mar 24;8:208. doi: 10.3389/fchem.2020.00208 (PMC7105854; doi:10.3389/fchem.2020.00208)
Supplement: Supplementary file 1 [file Data_Sheet_1.PDF]

Supplementary Material

1.- SYNTHESIS DETAILS

Synthesis of 1-(*n*-octyl)naphtalene (1)

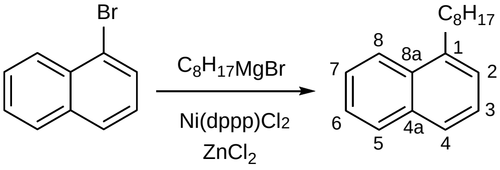

1

n-Octylmagnesium bromide (22 mmol) was added to a solution of $Ni(dppp)Cl_2$ (0.07 g, 1 mol %) and zinc chloride (2 g, 14 mmol) in dry THF (40 mL), and the reaction mixture was allowed to stir for 15 min in an inert atmosphere. 1-Bromonaphthalene (2 mL, 14 mmol) was then slowly added, and the mixture was heated at reflux overnight (65 °C). The end of the reaction was confirmed by t.l.c. (Hex:AcOEt 1:2). Saturated aqueous ammonium chloride (90 mL) was then added, and the solution was extracted with hexane (3x30 mL). The combined organic fractions were washed with H_2O (3x30 mL), dried with anhydrous $MgSO_4$, filtered and concentrated via rotary evaporation. The resulting product was dried under high vacuum (2×10^{-1} Pa) to obtain 1-(*n*-octyl)naphtalene (**1**) (3.3 g, 94 %) as a pure pale yellow liquid.[1]

^{1}H NMR (400 MHz, $CDCl_3$): δ= 8.10 (d, $J_{H,H}$=8 Hz, 1H, H-8), 7.90 (d, $J_{H,H}$=8 Hz, 1H, H-5), 7.75 (d, $J_{H,H}$=8 Hz, 1H, H-4), 7.57-7.49 (m, 2H, H-6, H-7), 7.44 (dd, $J_{H,H}$=8 Hz, 6.8 Hz, 1H, H-3), 7.36 (d, $J_{H,H}$=6.8 Hz, 1H, H-2), 3.11 (t, $J_{H,H}$=7.2 Hz, 2H, H-1’), 1.84-1.76 (m, 2H, H-2’), 1.49-1.31 (m, 10H, $(C\underline{H}_2)_5$), 0.95 (t, $J_{H,H}$=7.2 Hz, 3H, CH_3); **^{13}C NMR (100 MHz, $CDCl_3$):** δ= 139.15, 134.04, 132.06, 128.88, 126.53, 125.96, 125.73, 125.67, 125.47, 124.04, 33.31, 32.10, 31.05, 30.05, 29.72, 29.52, 22.88, 14.31.

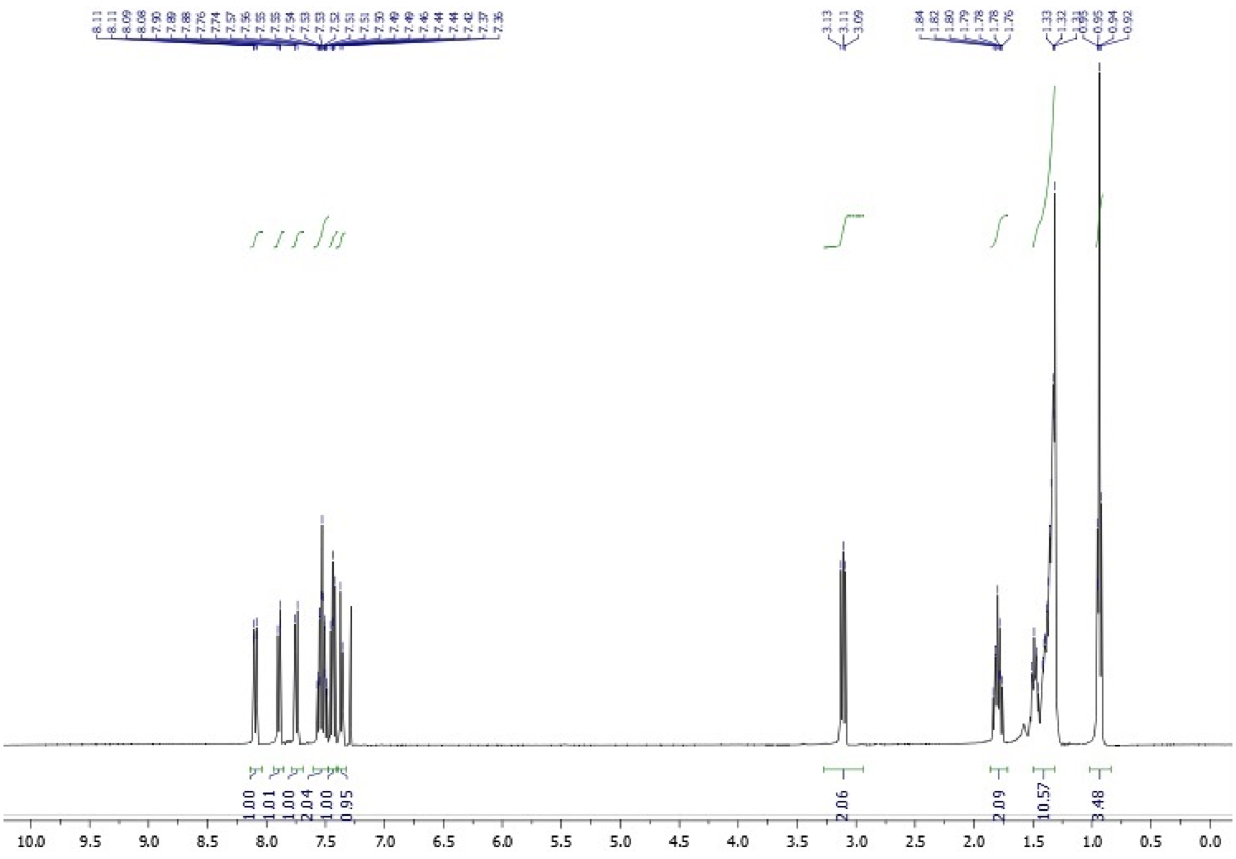

Figure S1. ^{1}H NMR spectrum of 1-(*n*-octyl)naphtalene (**1**)

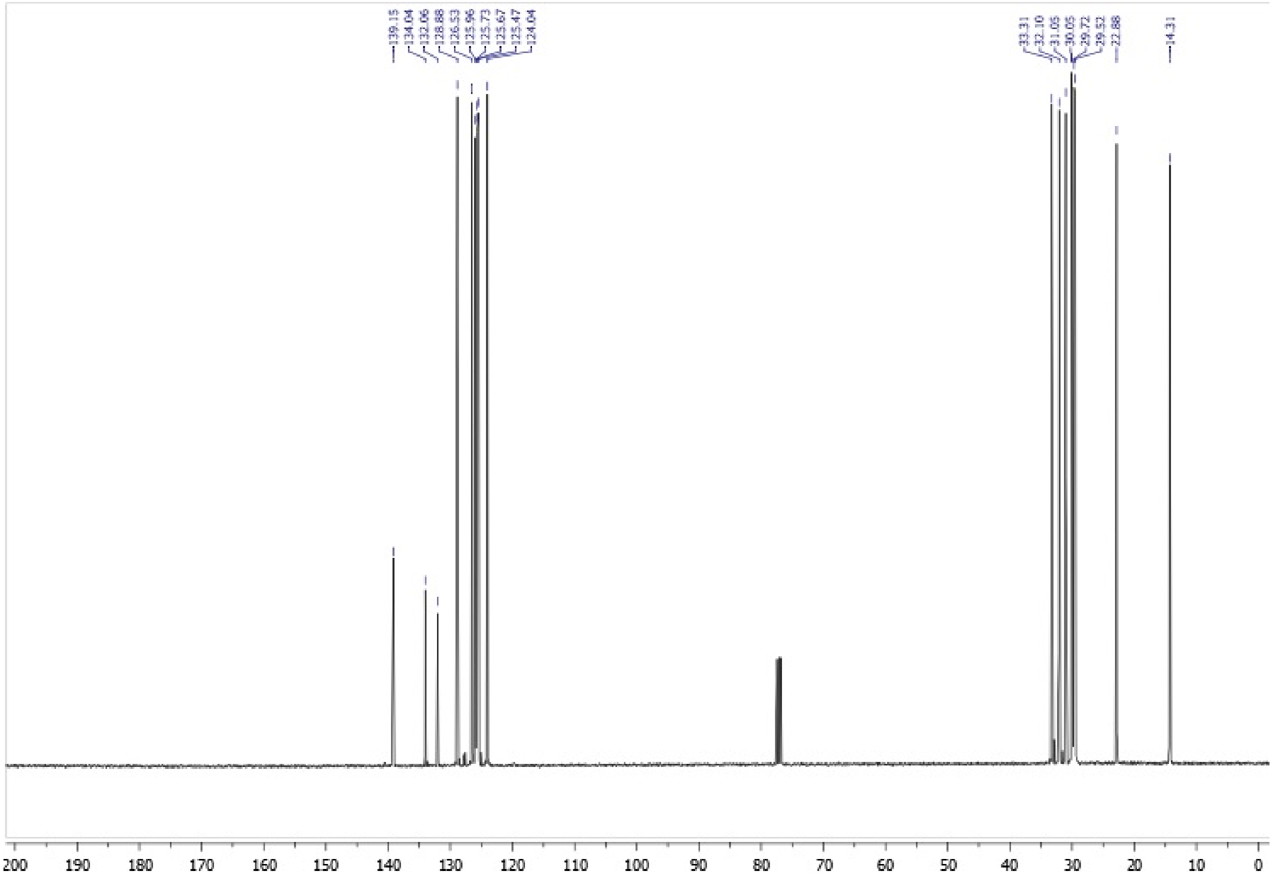

Figure S2. ^{13}C NMR spectrum of 1-(*n*-octyl)naphtalene (**1**)

Synthesis of sodium 4-(*n*-octyl)naphthalene-1-sulfonate Na[ONS] (2)

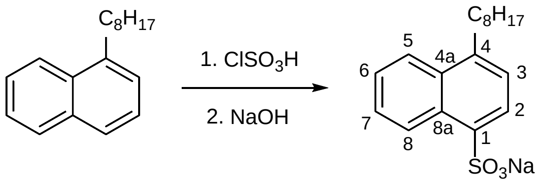

1 2

1-(*n*-octyl)naphtalene (**1**) (1.71 g, 7 mmol) was dissolved in chloroform (15 mL) and cooled to 0ºC. Chlorosulfonic acid (0.70 mL, 8.4 mmol) was added slowly to the solution and the mixture was stirring for 2 h. The reaction was neutralized by adding satured aqueous NaOH. The crude product was recrystallized in an ethanol/water mixture (50:50, vol/vol), filtered and dried under high vacuum (2 x 10^{-1} Pa) to obtain ONS (**2**) (2.2 g, 89 %) as white crystals.[2] Water content < 4900 ppm.

^{1}H NMR (400 MHz, D_2O): δ= 8.39 (d, $J_{H,H}$=7.3 Hz, 1H, H-8), 7.69 (d, $J_{H,H}$=7.2 Hz, 1H, H-5), 7.32 (d, $J_{H,H}$=8.1 Hz, 1H, H-2), 7.05 (t, $J_{H,H}$=7.2 Hz, 1H, H-7), 6.64 (m, 2H, H-6, H-3), 2.34 (m, 2H, H-1'), 1.35 (m, 2H, H-2'), 1.10-1.05 (m, 10H, $(C\underline{H_2})_5$), 0.70 (t, $J_{H,H}$=6.8 Hz, 3H, $C\underline{H_3}$); **^{13}C NMR (100 MHz, D_2O):** δ= 142.28, 136.88, 131.69, 128.57, 126.62, 126.16, 125.78, 125.65, 123.98, 123.16, 32.77, 31.85, 30.26, 29.77, 29.37, 22.59, 13.85.

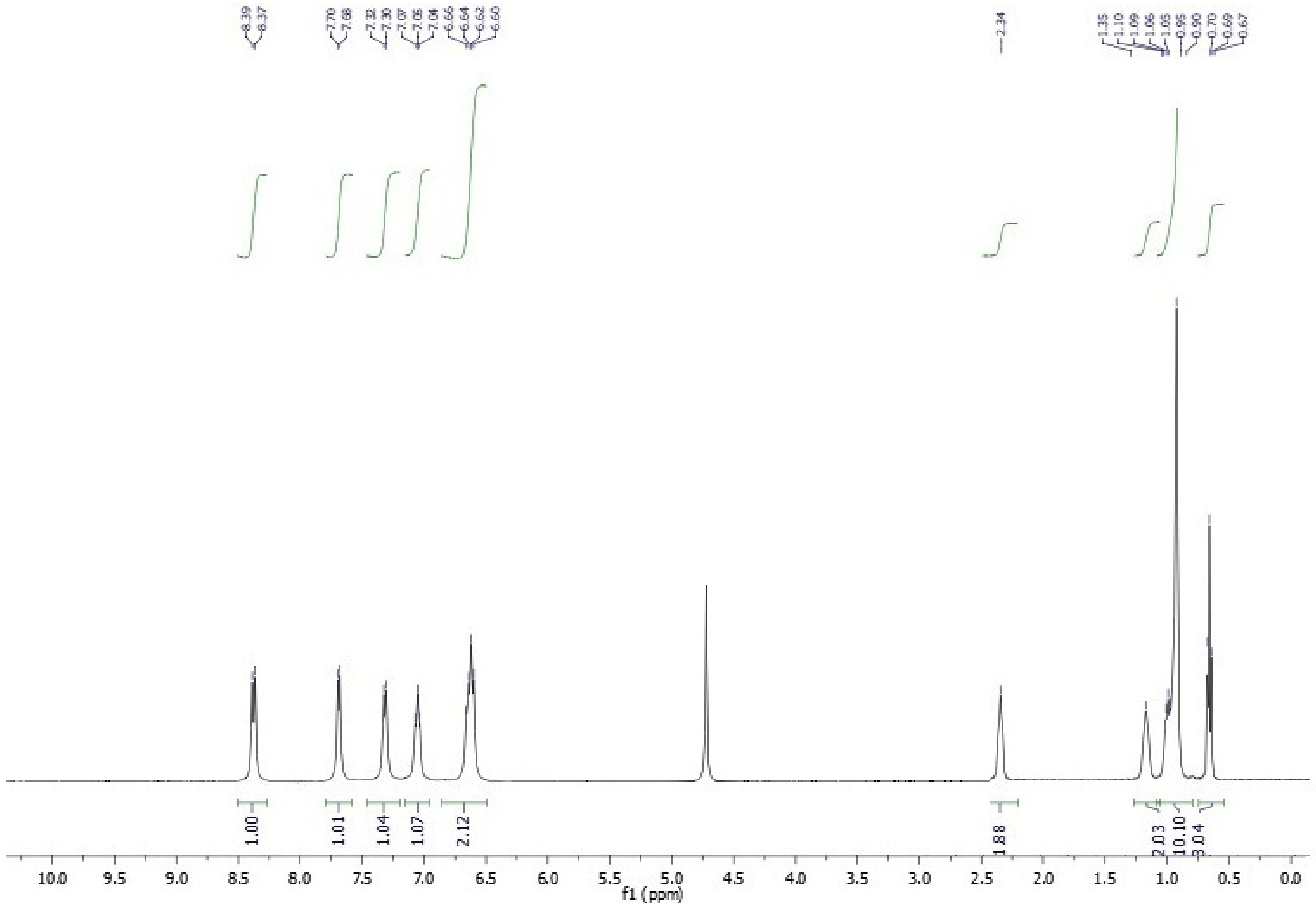

Figure S3. ^{1}H NMR spectrum of Na[ONS] (**2**)

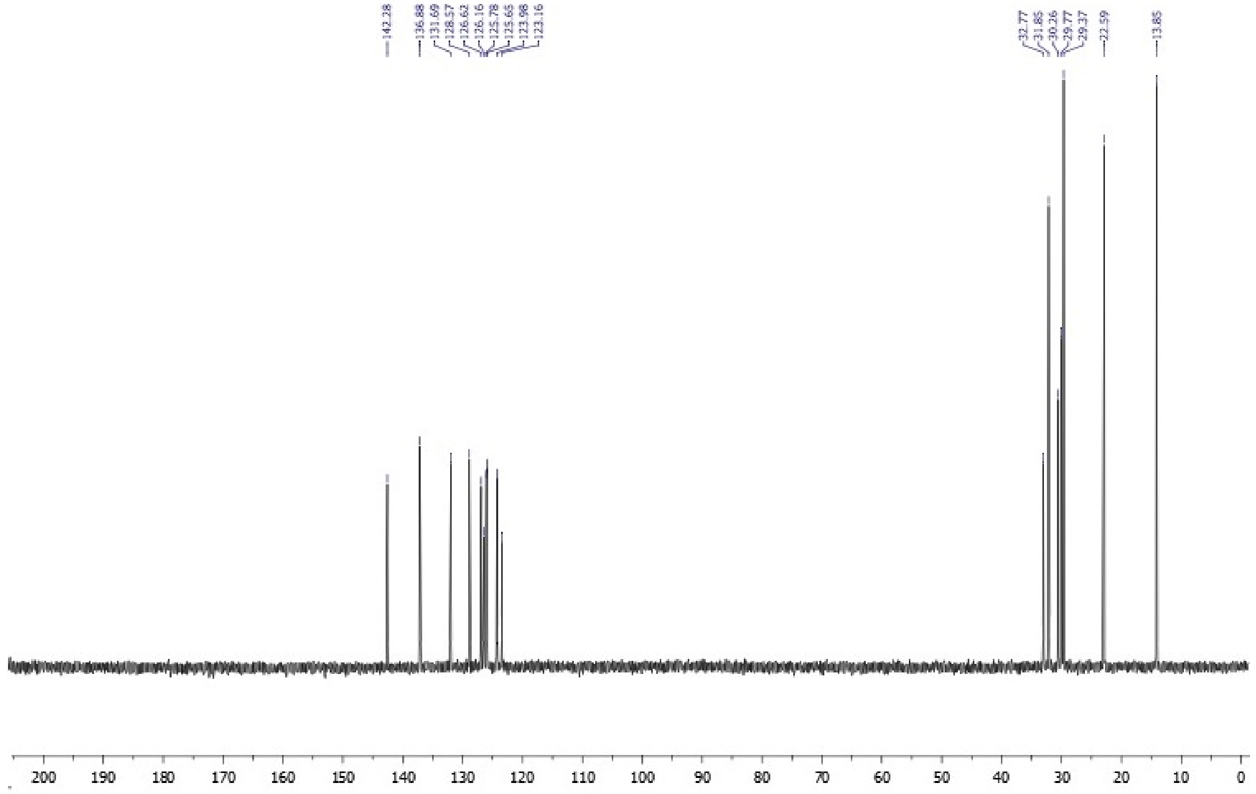

Figure S4. ^{13}C NMR spectrum of Na[ONS] (**2**)

Synthesis of α-(*n*-octyl)naphthalene sulfonate based ILs:

Metathesis general procedure (3-6): A mixture of the corresponding ILs $[C_1Pyr][H_2PO_4]$[3], $[C_1C_6Im]Cl$,[4] $[C_2Py]Br$,[5] or $[C_4Py]Cl$[5] and an equimolar amount of Na[ONS] (**2**) was heated at 120 °C and stirred for 30-60 min keeping the temperature.[6] After cooling down to r.t., the resulting reaction product was dissolved in CH_2Cl_2 to precipitate the inorganic formed salt, which was filtered off. The filtrate was concentrated and dried under high vacuum (2 x 10^{-1} Pa) to afford 1-(*n*-octyl)naphthalene sulfonate ILs **3**-**6**.

Synthesis of N-methylpirrolidinium 4-(*n*-octyl)naphthalene-1-sulfonate $[C_1Pyr][ONS]$ (3)

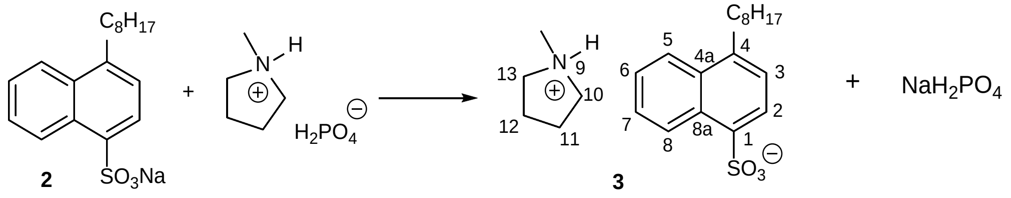

The general procedure was applied to obtain **3** (95%) as a yellow viscous liquid. Water content < 2100 ppm.

^{1}H NMR (400 MHz, $CDCl_3$): δ= 10.38 (s, 1H, NH), 8.91 (dd, $J_{H,H}$=8.3, 1.1 Hz 1H, H-8), 8.05 (m, 2H, H-2, H-5), 7.53-7.46 (m, 2H, H-6, H-7), 7.23 (d, $J_{H,H}$=7.4 Hz, 1H, H-3), 3.52 (m, 2H, H-10), 3.01 (t, $J_{H,H}$=7.7 Hz, 2H, H-1’), 2.69 (d, $J_{H,H}$= 4.9 Hz, 3H, $NC\underline{H}_3$), 2.65 (m, 2H, H-13), 1.86 (m, 4H, H-12, H-11), 1.67 (q, $J_{H,H}$=7.6 Hz 2H, H-2’), 1.32-1.24 (m, 10H, $(C\underline{H}_2)_5$), 0.88 (t, $J_{H,H}$= 6.8 Hz, 3H, $C\underline{H}_3$); **^{13}C NMR (100 MHz, $CDCl_3$):** 142.47, 138.93, 132.37, 130.19, 129.27, 127.16, 126.36, 125.88, 125.32, 124.35, 124.05, 55.39, 40.69, 33.30, 31.84, 30.78, 29.78, 29.42, 29.27, 22.99, 22.63, 14.10; **HRMS (ESI) *m/z*** (%): calcd for $[(C_5H_{12}N)_2(C_{18}H_{23}O_3S)]^+$: 491.3303 $[A_2B]^+$; found: 491.3302 (100); **ICP-MS**: 0.068 % Na.

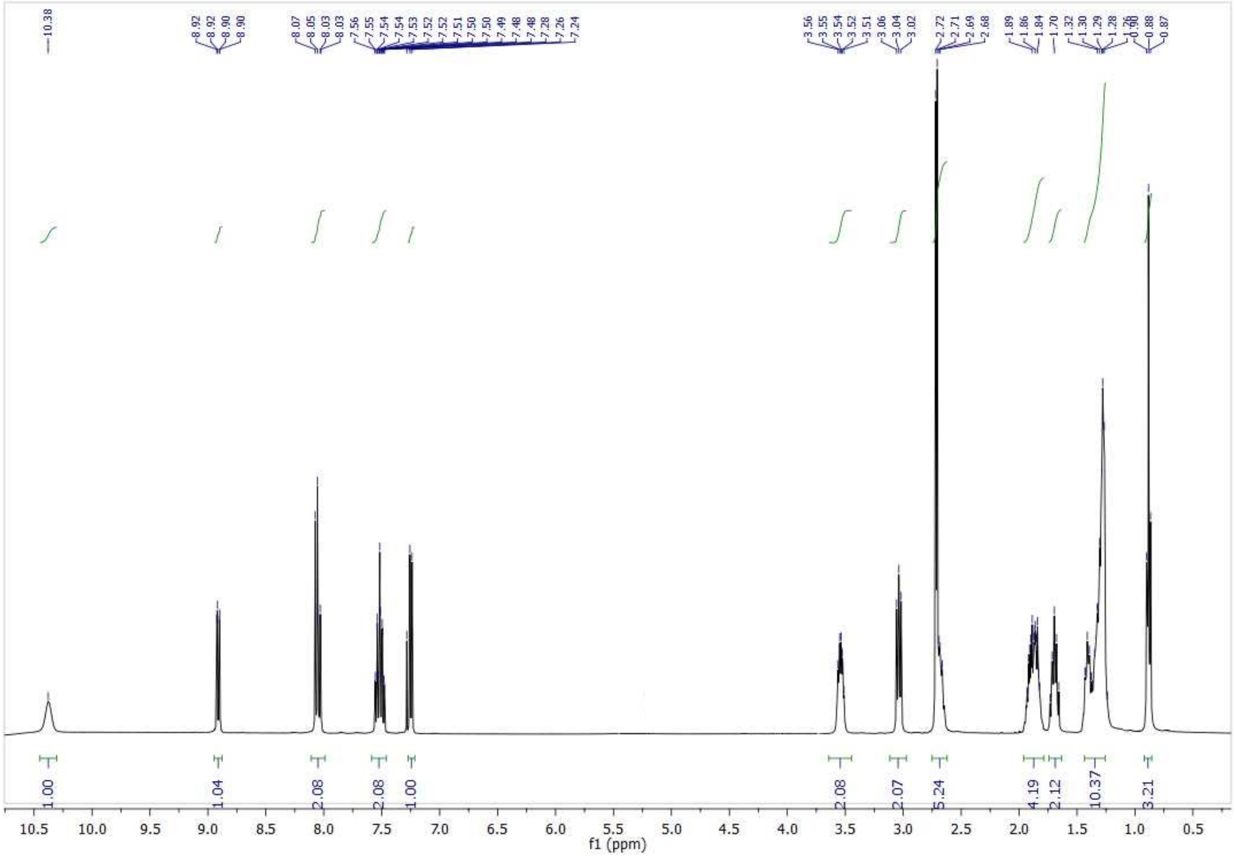

Figure S5. ^{1}H NMR spectrum of [C_1Pyr][ONS] (**3**)

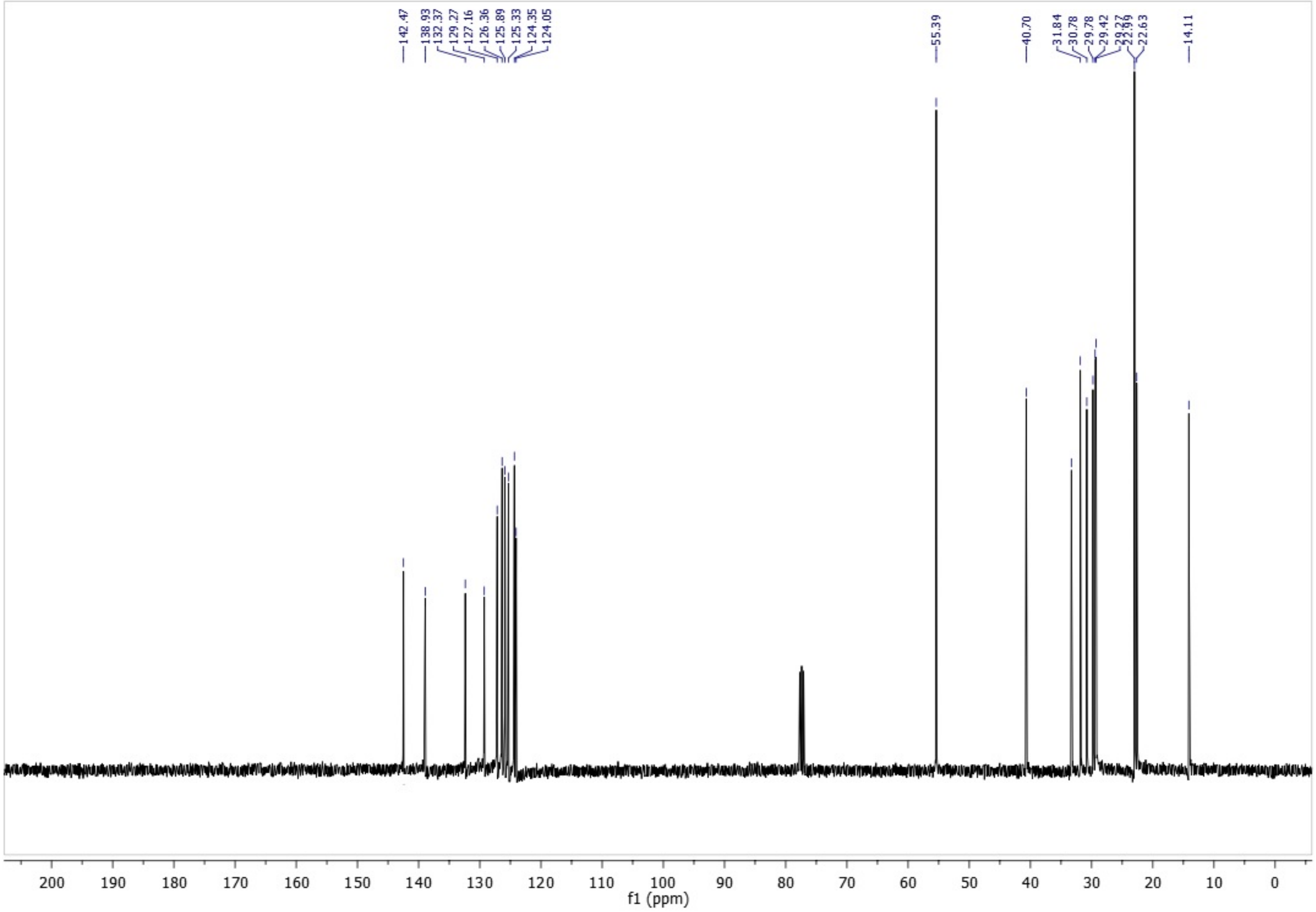

Figure S6. ^{13}C NMR spectrum of [C_1Pyr][ONS] (**3**)

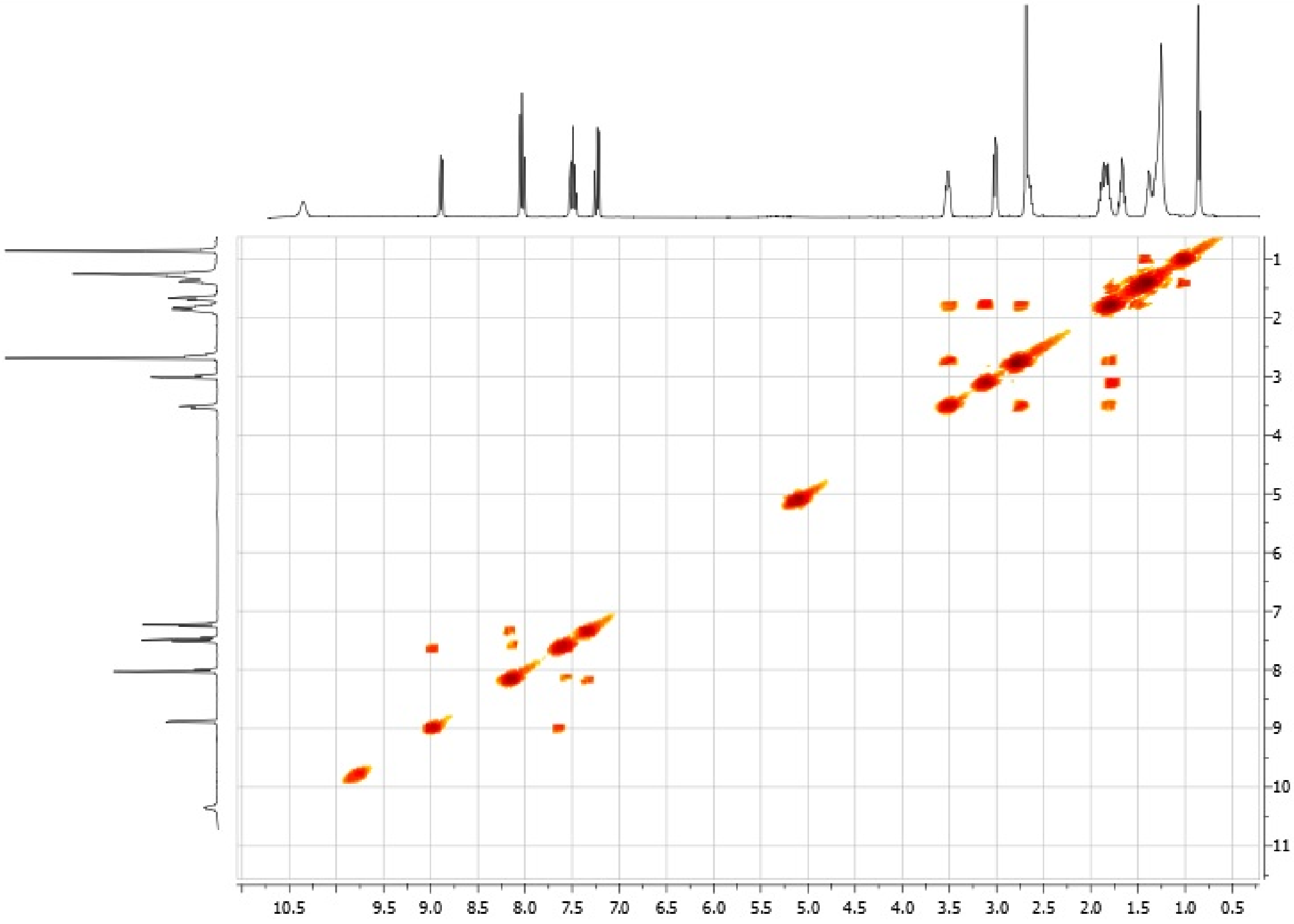

Figure S7. H-H COSYspectrum of [C_1Pyr][ONS] (**3**)

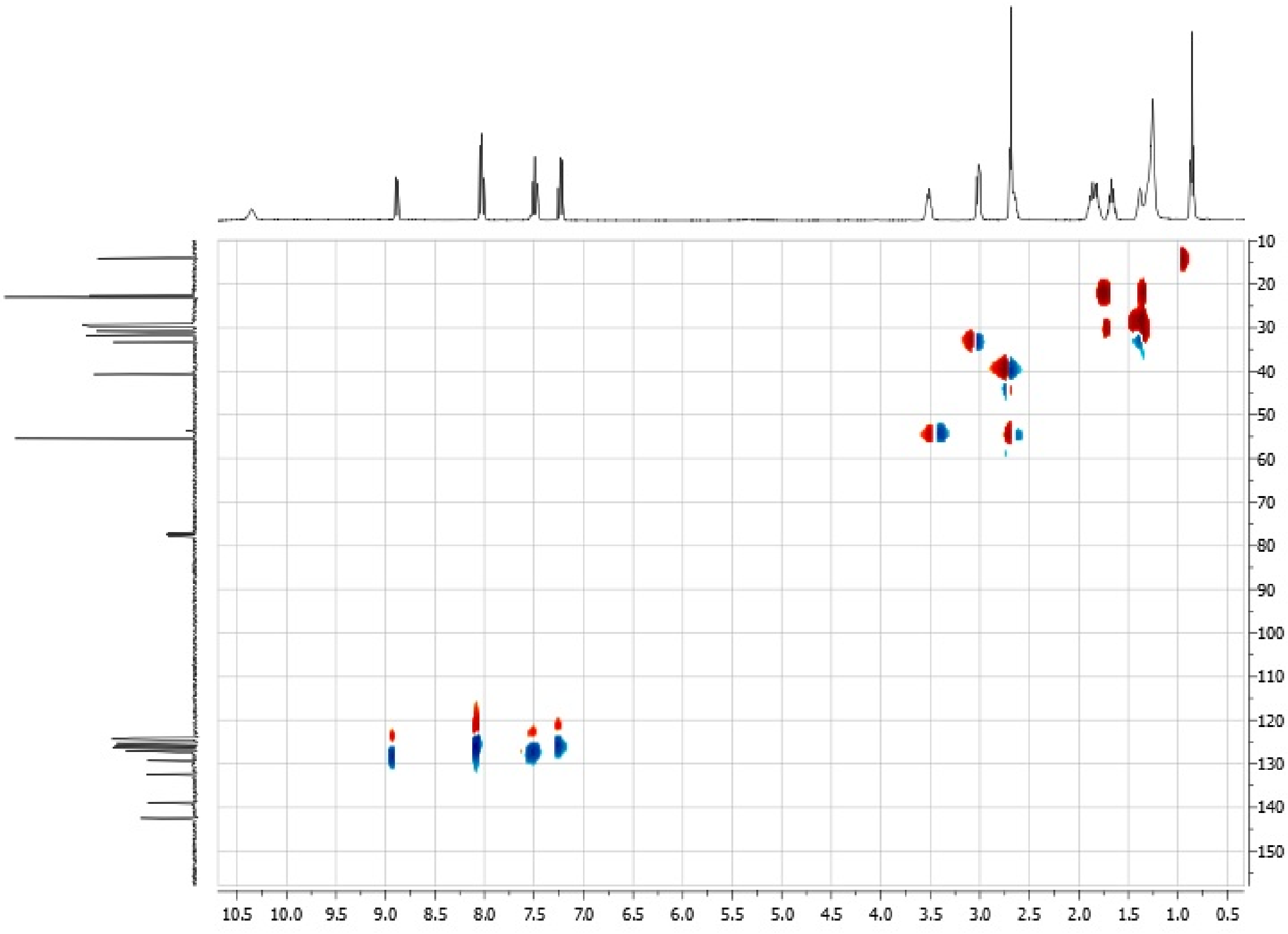

Figure S8. HSQC spectrum of [C_1Pyr][ONS] (**3**)

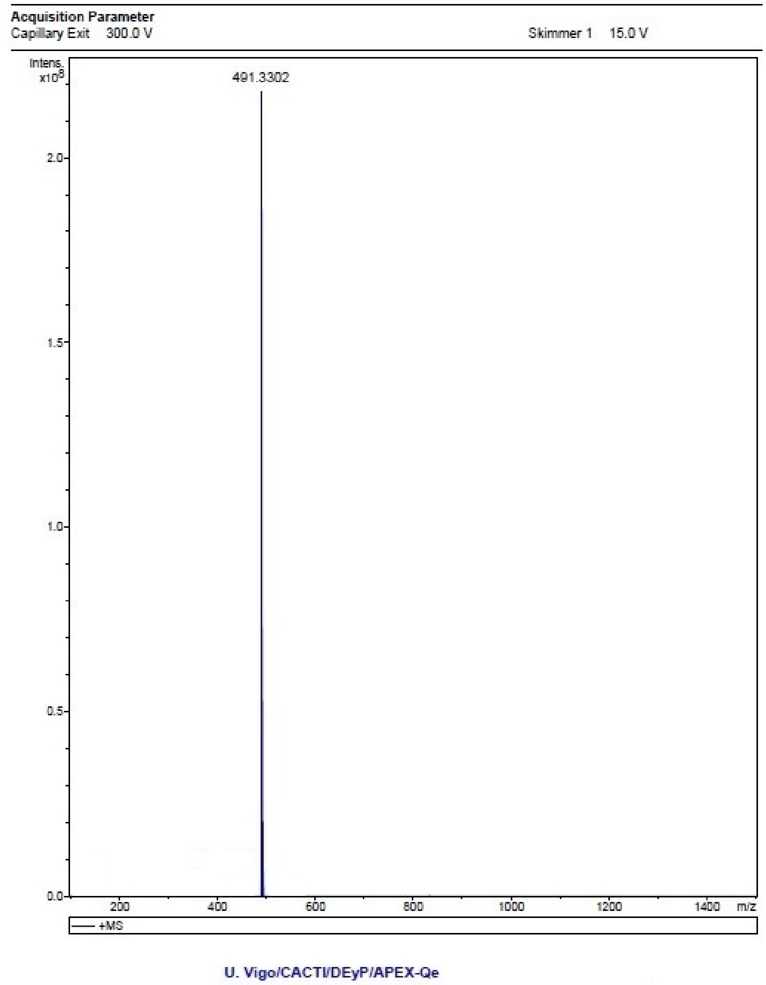

Mass Spectrum Molecular Formula Report

Meas. m/z	#	Ion Formula	m/z	err [ppm]	mSigma	# mSigma	Score	rdb	e⁻ Conf	N-Rule
491.3303	1	C28H47N2O3S	491.3302	-0.2	24.6	1	100.00	6.5	even	ok

Figure S9. HRMS spectrum of [C_1Pyr][ONS] (**3**)

Synthesis of 1-hexyl-3-methylimidazolium 4-(n-octyl)naphthalene-1-sulfonate [C_1C_6Im][ONS] (4)

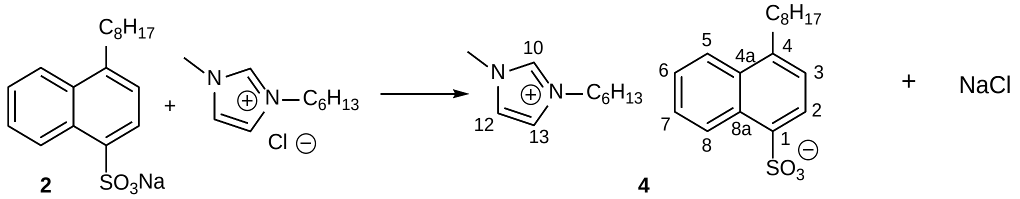

The general procedure was applied to obtain **4** (98 %) as a solid. Water content < 4200 ppm.

^{1}H NMR (400 MHz, $CDCl_3$): δ= 9.60 (s, 1H, H-10), 9.02 (m, 1H, H-8), 8.11 (d, $J_{H,H}$=7.4 Hz, 1H, H-12), 8.02 (m, 1H, H-5), 7.49 (m, 2H, H-6, H-7), 7.30 (m, 1H, H-2), 7.23 (d, J= 7.4 Hz, 1H, H-13), 7.14 (m, 1H, H-3), 3.94 (t, J=7.5 Hz, 2H, $NC\underline{H_2}(CH_2)_4$), 3.82 (s, 3H, $NC\underline{H_3}$), 3.03 (t, J=7.8 Hz, 2H, H-1'), 1.73-1.56 (m, 4H, H-2', $NCH_2C\underline{H_2}$), 1.44-1.28 (m, 10H, $(C\underline{H_2})_5$), 1.13 (m, 6H, $N(CH_2)_2(C\underline{H_2})_3$), 0.90 (t, J=6.8 Hz, 3H, $N(C_5H_{10})C\underline{H_3}$), 0.83 (t, $J_{H,H}$=7 Hz, 3H, $(C_7H_{14})C\underline{H_3}$) ; **^{13}C NMR (100 MHz, $CDCl_3$):** 141.64, 140.53, 137.07, 132.38, 129.58, 127.68, 125.92, 125.58, 125.09, 124.28, 123.88, 123.64, 121.93, 49.56, 36.02, 33.30, 31.83, 30.94, 30.79, 29.87, 29.79, 29.43, 29.27, 25.64, 22.61, 22.27, 14.07, 13.87; **HRMS (ESI)** ***m/z*** (%): calcd for $[(C_{10}H_{19}N_2)_2(C_{18}H_{23}O_3S)]^+$: 653.4471 $[A_2B]^+$; found: 653.4459 (100). **ICP-MS**: 0.004 % Na, 0.006 % Cl.

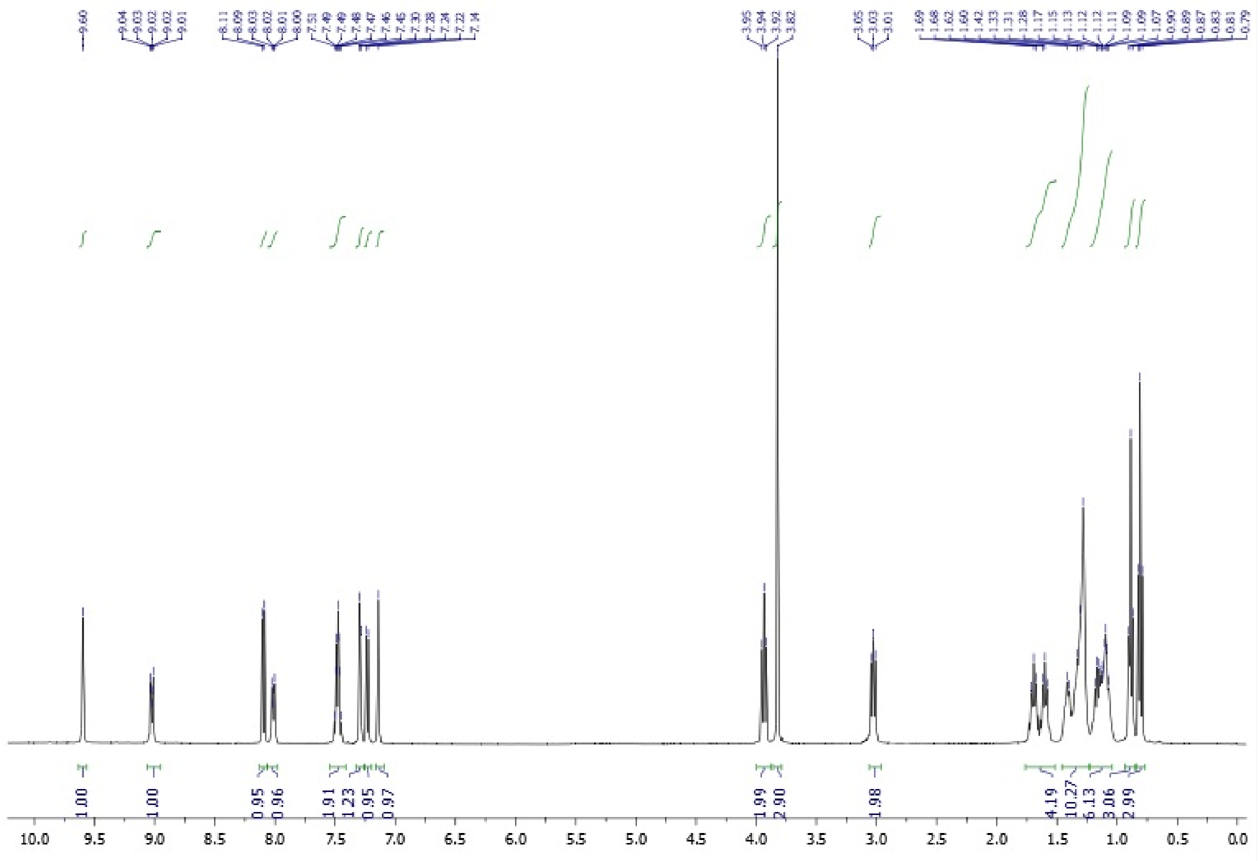

Figure S10. ^{1}H NMR spectrum of $[C_1C_6Im][ONS]$ (**4**)

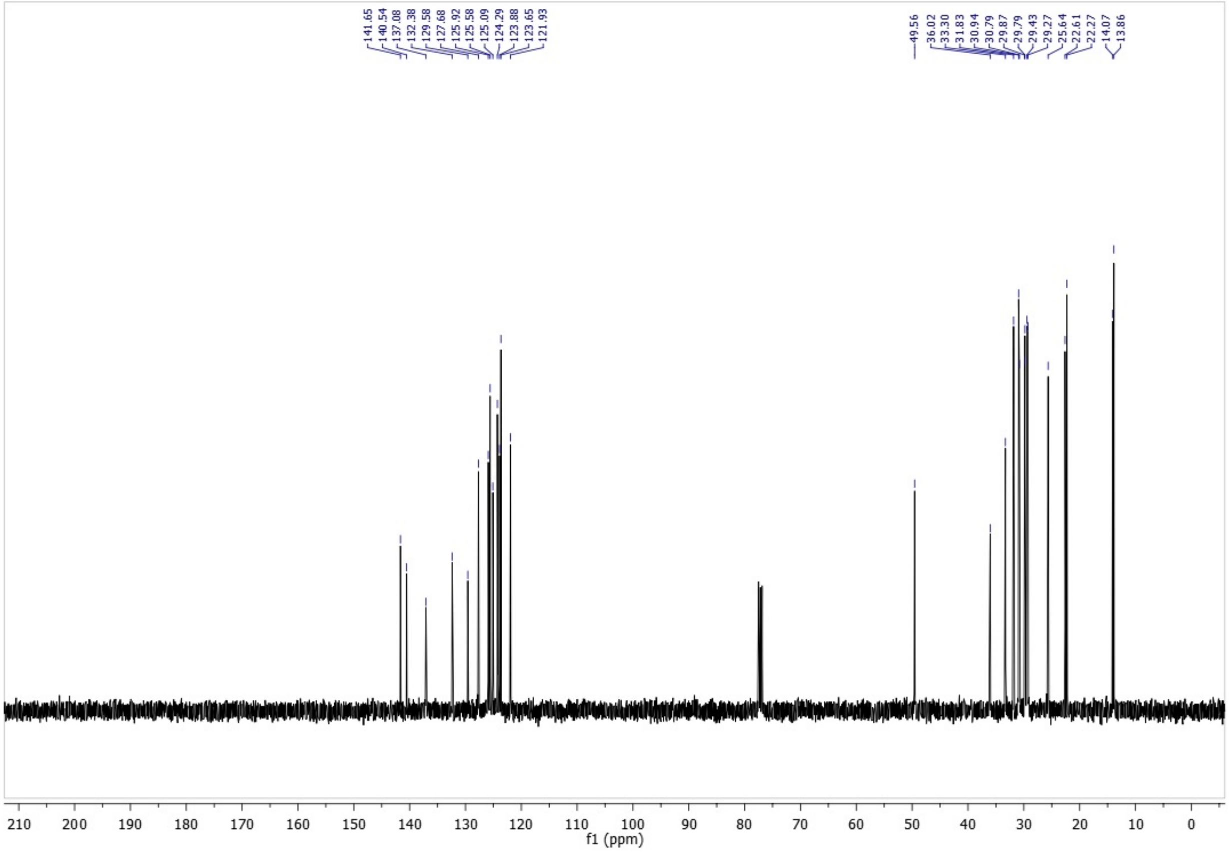

Figure S11. ^{13}C NMR spectrum of [C_1C_6Im][ONS] (**4**)

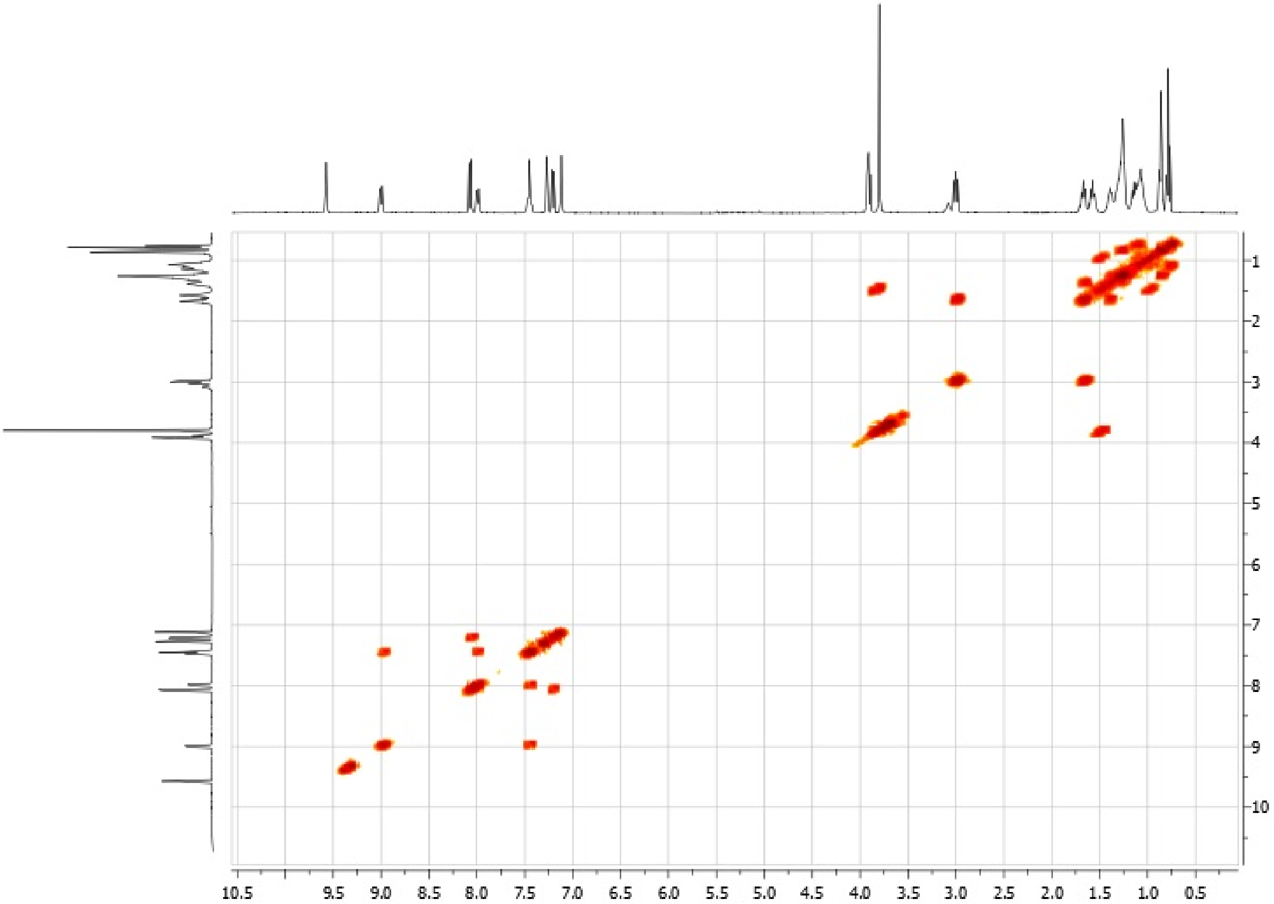

Figure S12. H-H COSY spectrum of [C_1C_6Im][ONS] (**4**)

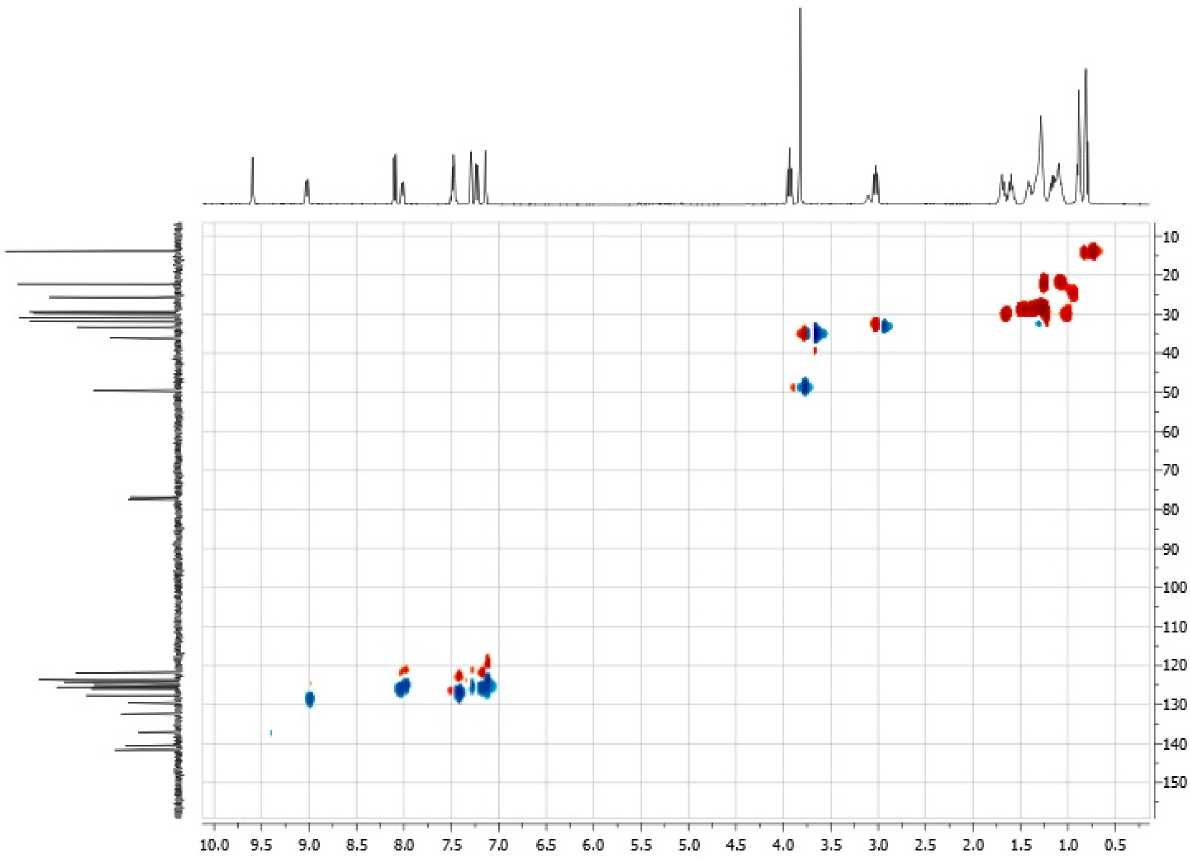

Figure S13. HSQC spectrum of [C_1C_6Im][ONS] (**4**)

Acquisition Parameter
Capillary Exit 300.0 V Skimmer 1 15.0 V

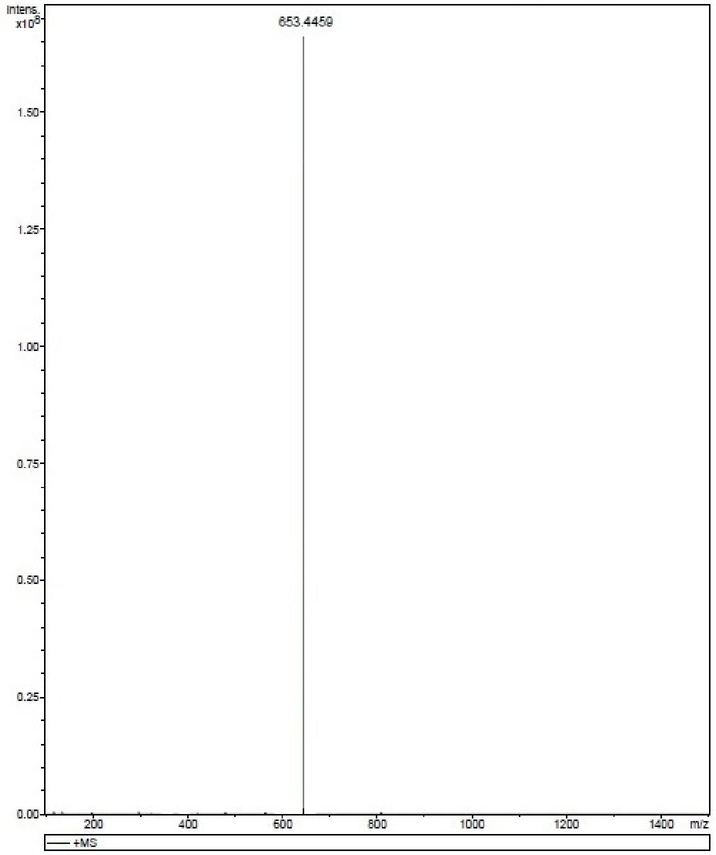

U. Vigo/CACTI/DEyP/APEX-Qe

Mass Spectrum Molecular Formula Report

Meas. m/z	#	Ion Formula	m/z	err [ppm]	mSigma	# mSigma	Score	rdb	e⁻ Conf	N-Rule
653.4471	1	C38H61N4O3S	653.4459	-1.9	n.a.	3	54.76	10.5	even	ok

Figure S14. HRMS spectrum of [C_1C_6Im][ONS] (**4**)

Synthesis of 1-ethylpyridinium 4-(*n*-octyl)naphthalene-1-sulfonate [C_2Py][ONS] (5)

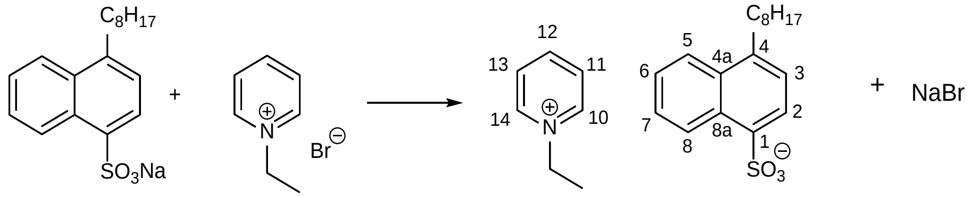

2 5

The general procedure was applied to obtain **5** (98%) as a solid. Water content < 3500 ppm.

^{1}H NMR (400 MHz, $CDCl_3$): δ= 9.12 (d, $J_{H,H}$=5.8 Hz, 2H, H-14, H-10), 8.98 (m, 1H, H-8), 8.17 (t, $J_{H,H}$=7.7 Hz, 1H, H-12), 8.09 (d, $J_{H,H}$= 7.4 Hz, 1H, H-5), 8.01 (m, 1H, H-2), 7.79 (t, $J_{H,H}$=6.8 Hz 2H, H-13, H-11), 7.47 (m, 2H, H-7, H-6), 7.20 (d, J=7.4 Hz, 1H, H-3), 4.70 (m, 2H, $NC\underline{H_2}$), 3.01 (m, 2H, H-1'), 1.68 (m, 2H, H-2'), 1.46 (t, $J_{H,H}$=7.3 Hz, 3H, $NCH_2C\underline{H_3}$), 1.42-1.25 (m, 10H, $(C\underline{H_2})_5$), 0.89 (t, $J_{H,H}$=7 Hz, 3H, $C\underline{H_3}$) ; **^{13}C NMR (100 MHz, $CDCl_3$):** 144.79, 144.50, 142.00, 140.01, 132.38, 129.49, 128.27, 127.55, 126.27, 125.82, 125.38, 124.51, 124.00, 57.26, 33.35, 31.90, 30.92, 29.88, 29.72, 29.49, 29.35, 22.68, 16.76, 14.14; **HRMS (ESI) *m/z*** (%)*:* calcd for $[(C_7H_{10}N)_2(C_{18}H_{23}O_3S)]^+$: 535.2999 $[A_2B]^+$; found: 535.2989 (100). **ICP-MS**: 0.164 % Na, 0.113 % Br.

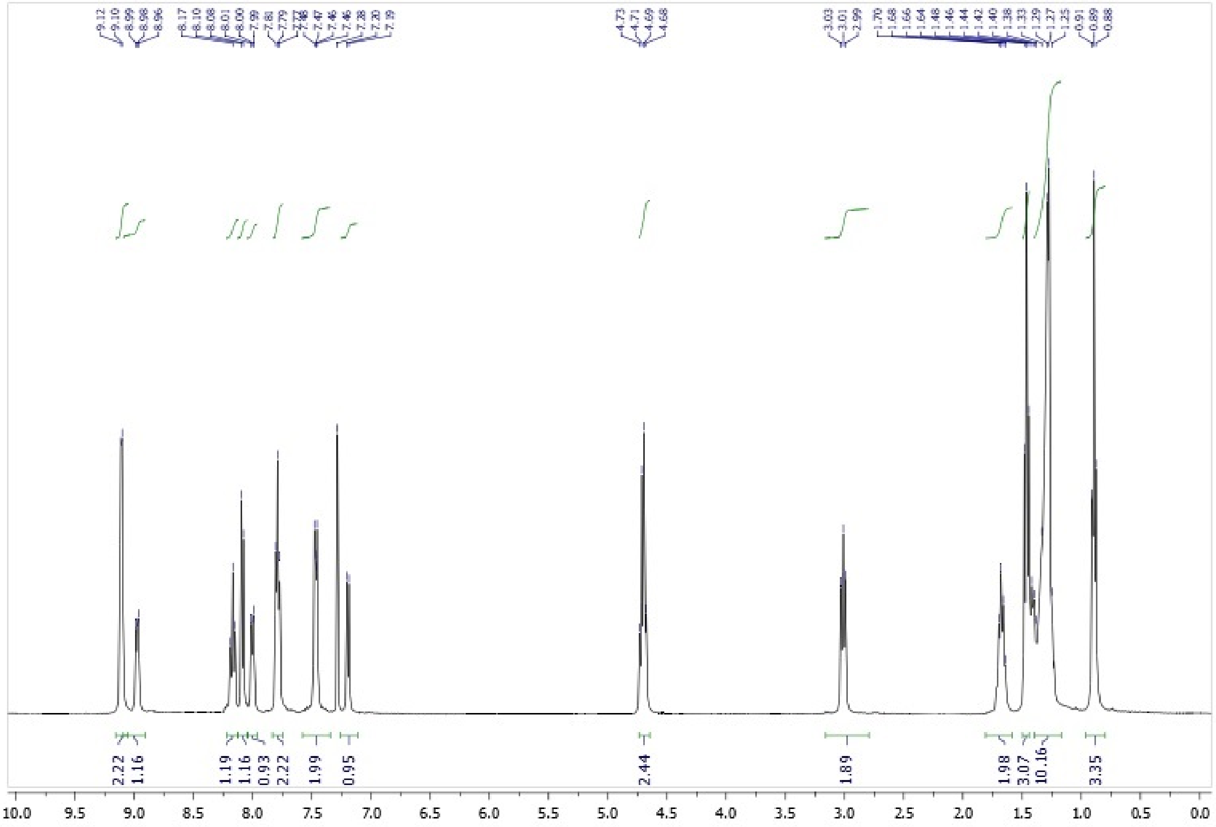

Figure S15. ^{1}H NMR spectrum of [C_2Py][ONS] (**5**)

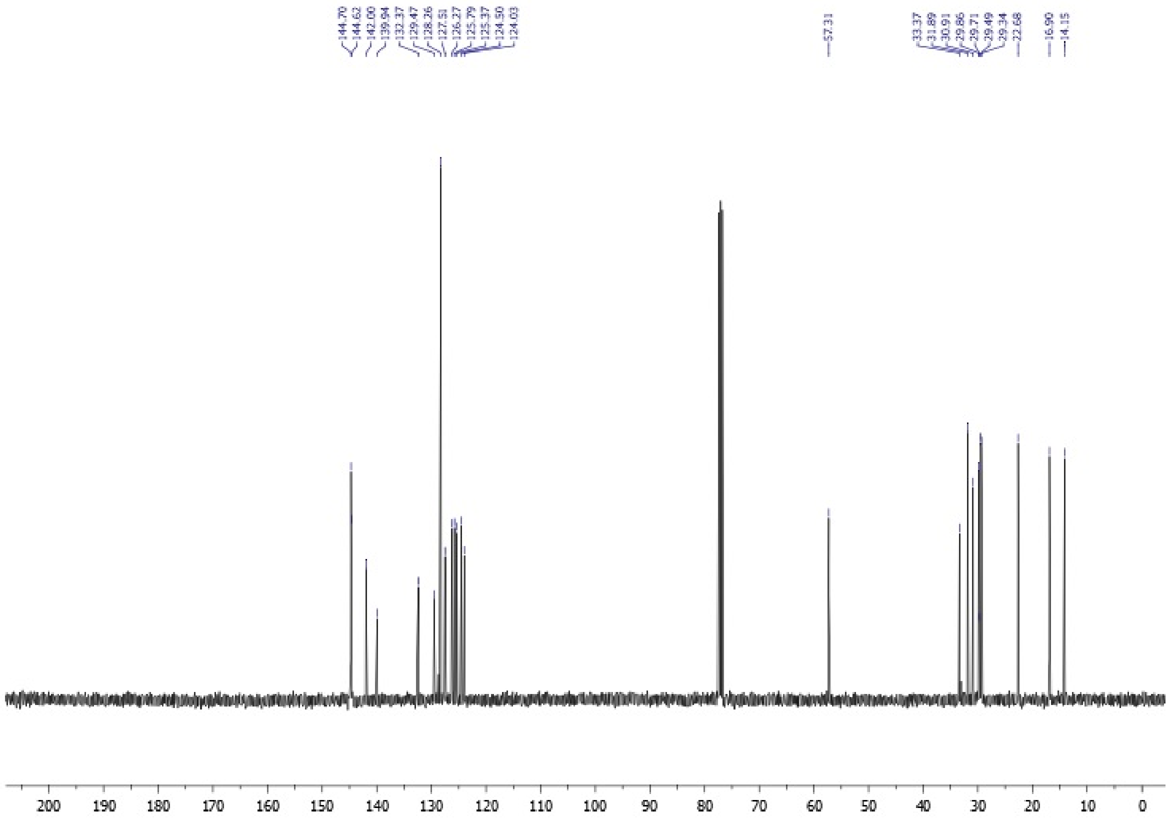

Figure S16. ^{13}C NMR spectrum of [C_2Py][ONS] (**5**)

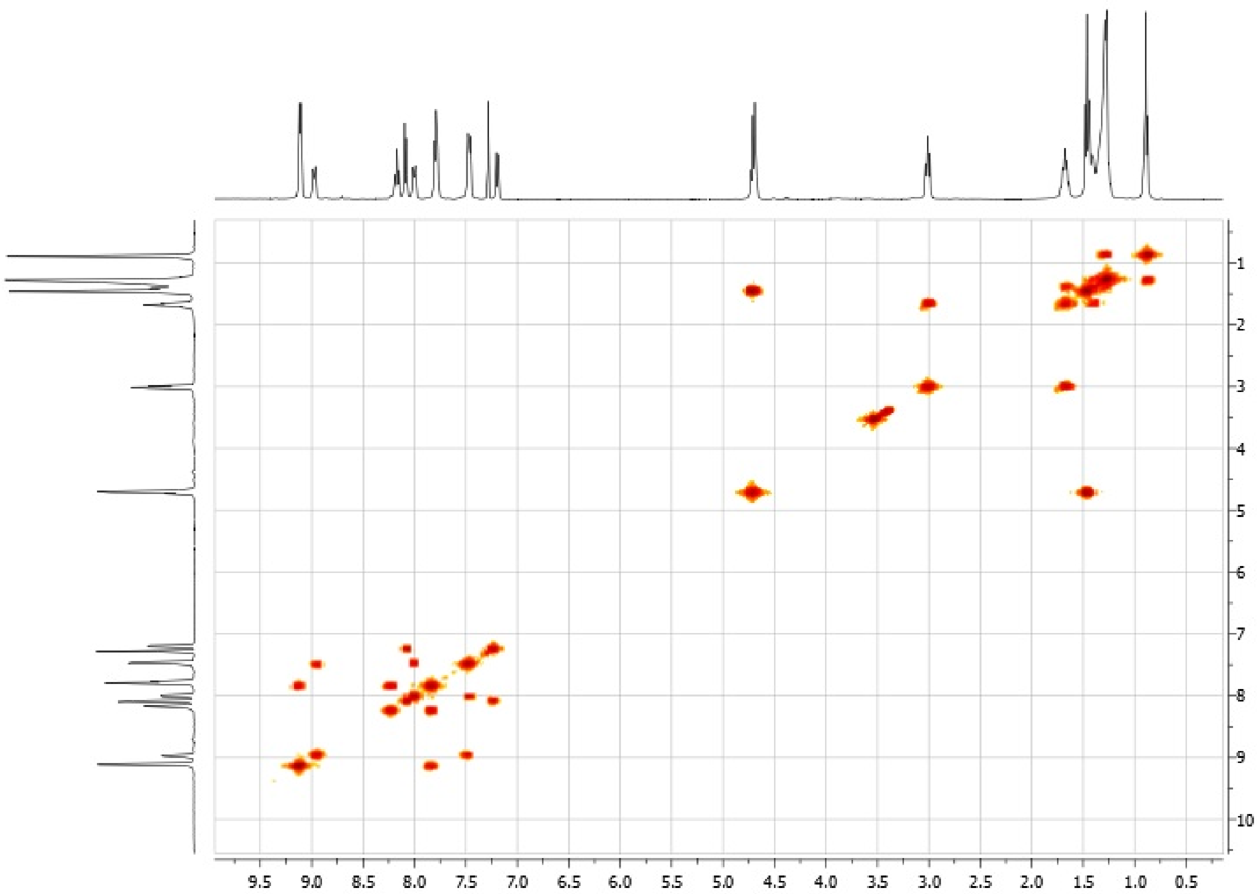

Figure S17. H-H COSY spectrum of [C_2Py][ONS] (**5**)

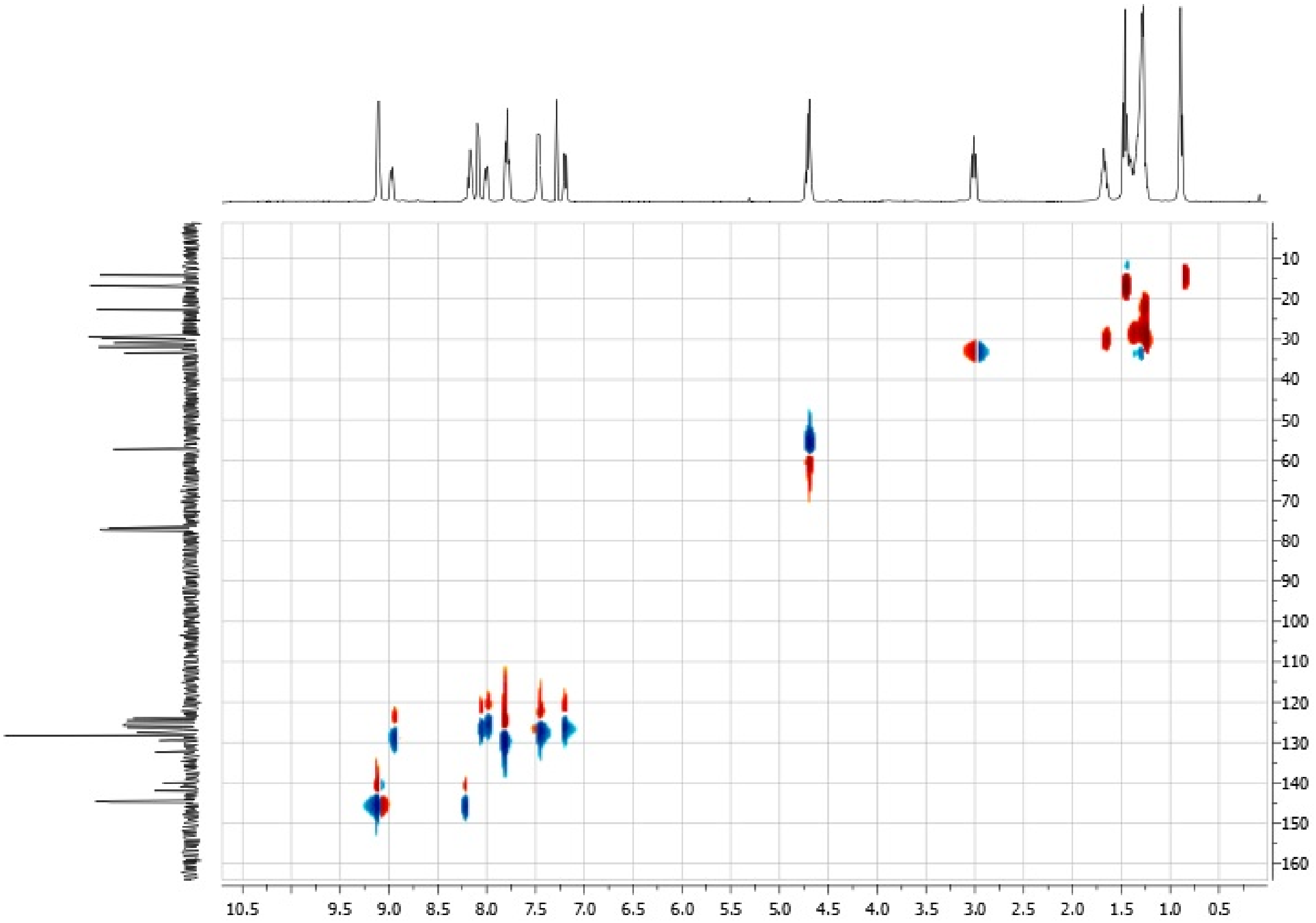

Figure S18. HSQC spectrum of [C_2Py][ONS] (**5**)

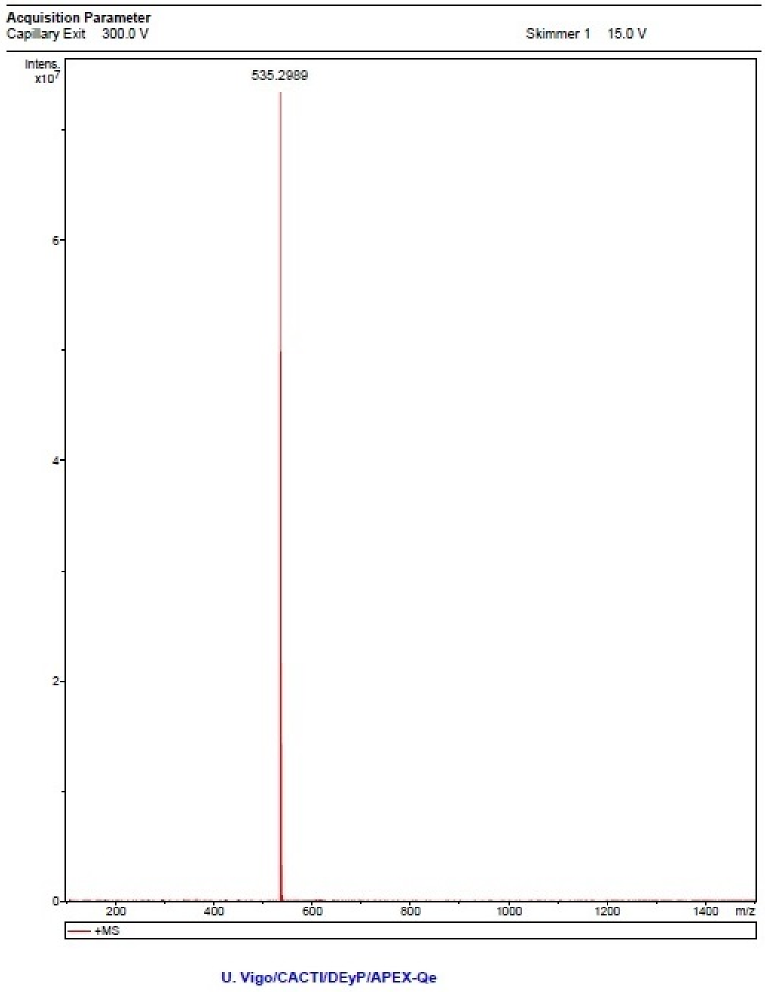

Mass Spectrum Molecular Formula Report

Meas. m/z	#	Ion Formula	m/z	err [ppm]	mSigma	# mSigma	Score	rdb	e⁻ Conf	N-Rule
535.2999	1	C32H43N2O3S	535.2989	-1.9	69.7	2	34.86	12.5	even	ok

Figure S19. HRMS spectrum of [C_2Py][ONS] (**5**)

Synthesis of 1-butylpyridinium 4-(n-octyl)naphthalene-1-sulfonate [C_4Py][ONS] (6)

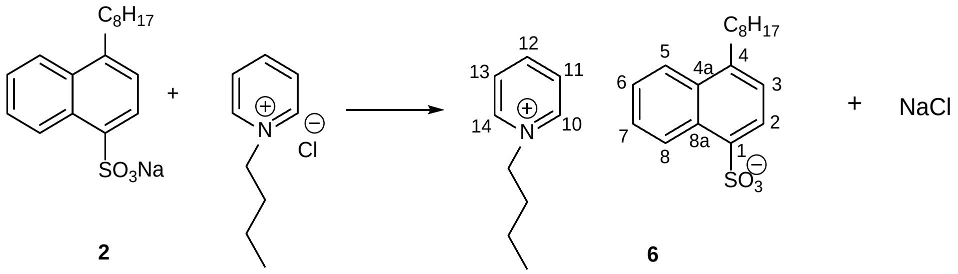

2 **6**

The general procedure was applied to obtain **6** (97%) as a solid. Water content < 1200 ppm.

^{1}H NMR (400 MHz, $CDCl_3$): δ= 9.09 (m, 2H, H-14, H-10), 9.02 (m, 1H, H-8), 8.25 (t, J=7.7 Hz, 1H, H-12), 8.10 (d, J= 7.4 Hz, 1H, H-5), 8.02 (m, 1H, H-2), 7.88 (m, 2H, H-13, H-11), 7.50 (m, 2H, H-6, H-7), 7.25 (d, J= 7.4 Hz, 1H, H-3), 4.65 (m, 2H, $NC\underline{H_2}(CH_2)_2$), 3.04 (m, 2H, H-1'), 1.80-1.68 (m, 4H, $NCH_2C\underline{H_2}$, H-2'), 1.42-1.27 (m, 10H, $(C\underline{H_2})_5$), 1.18 (m, 2H, $N(CH_2)_2C\underline{H_2}CH_3$), 0.90 (t, J= 6.8 Hz, 3H, $C\underline{H_3}$), 0.80 (t, J=7.3 Hz, 3H, $N(CH_2)_3C\underline{H_3}$); **^{13}C NMR (100 MHz, $CDCl_3$):** 144.85, 144.76, 141.89, 140.22, 132.40, 129.55, 128.25, 127.65, 126.16, 125.76, 125.32, 124.44, 123.97, 61.56, 33.33, 33.29, 31.88, 30.89, 29.84, 29.70, 29.448, 29.32, 22.66, 19.07, 14.12, 13.32; **HRMS (ESI) *m/z* (%):** calcd for $[(C_9H_{14}N)_2(C_{18}H_{23}O_3S)]^+$: 591.3596 $[A_2B]^+$; found: 591.3615 (100). **ICP-MS**: 0.078% Na, 0.085% Cl.

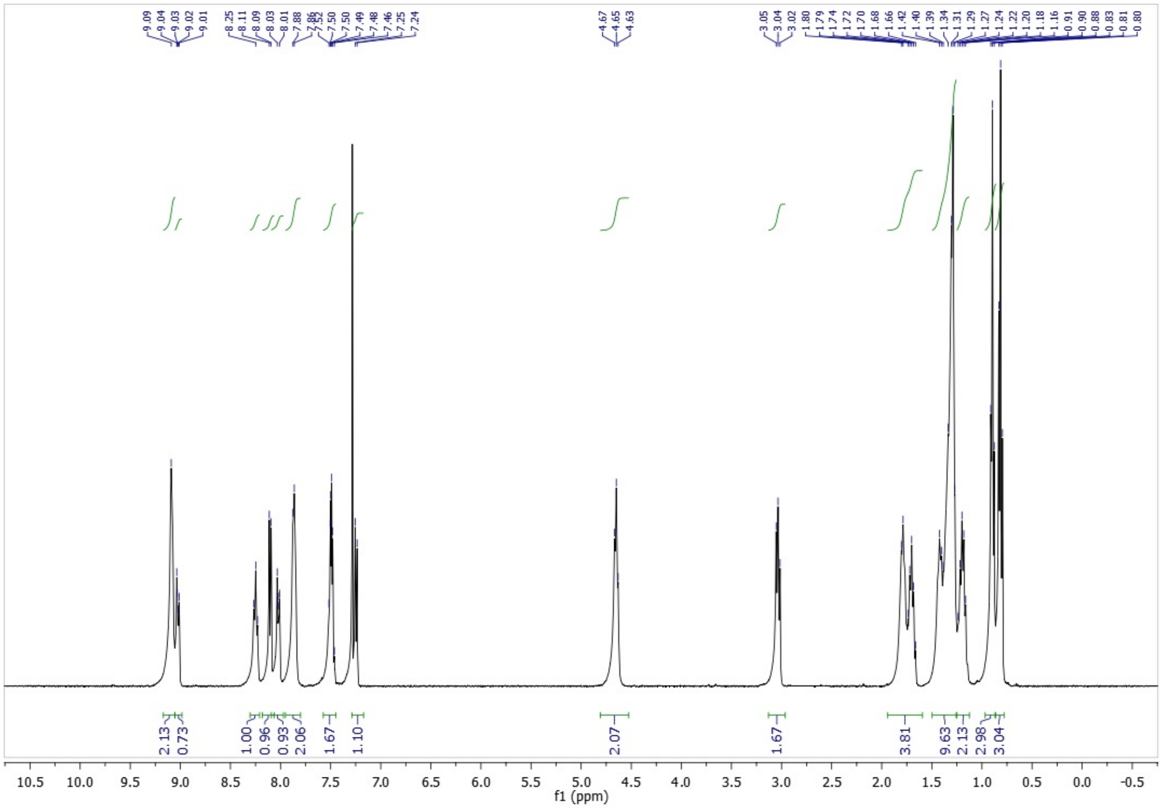

Figure S20. ^{1}H NMR spectrum of [C_4Py][ONS] (**6**)

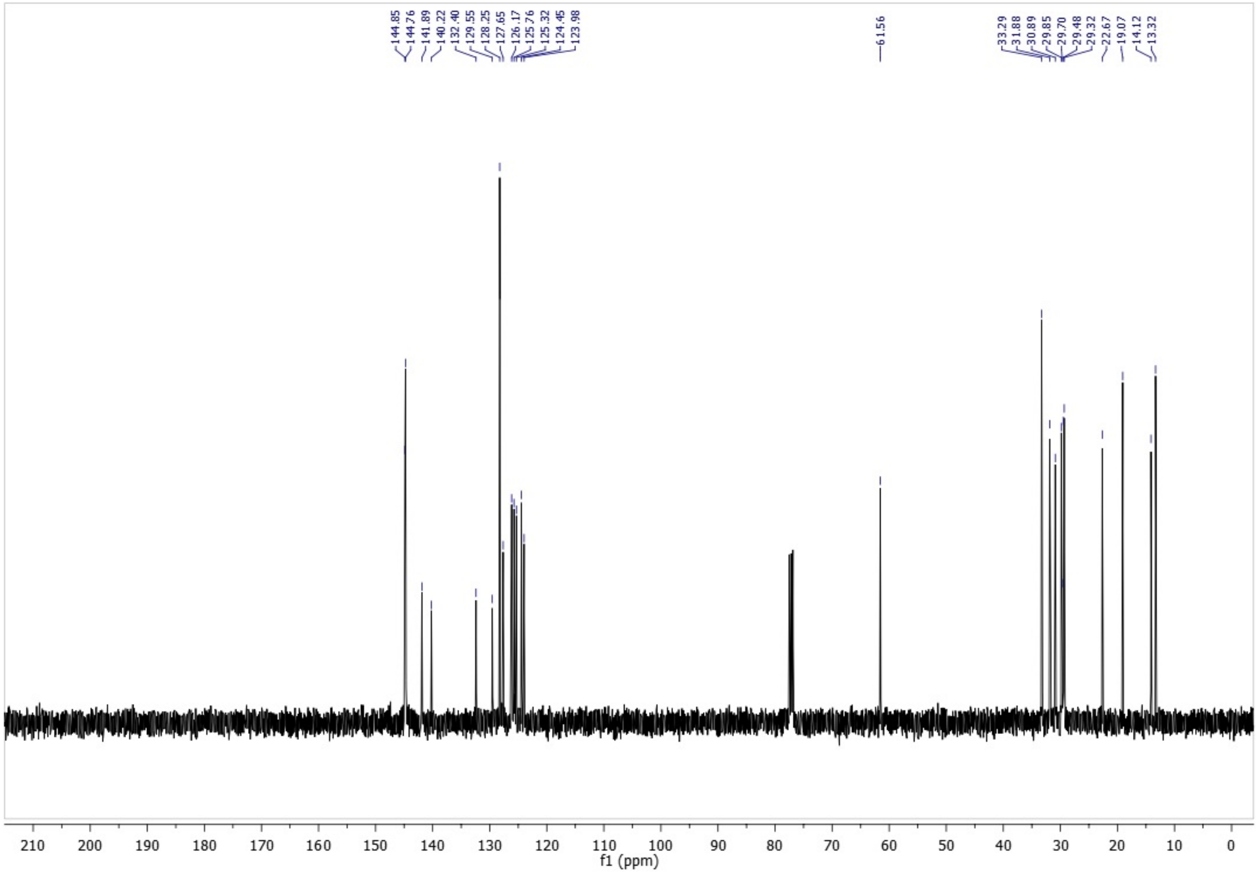

Figure S21. ^{13}C NMR spectrum of [C_4Py][ONS] (**6**)

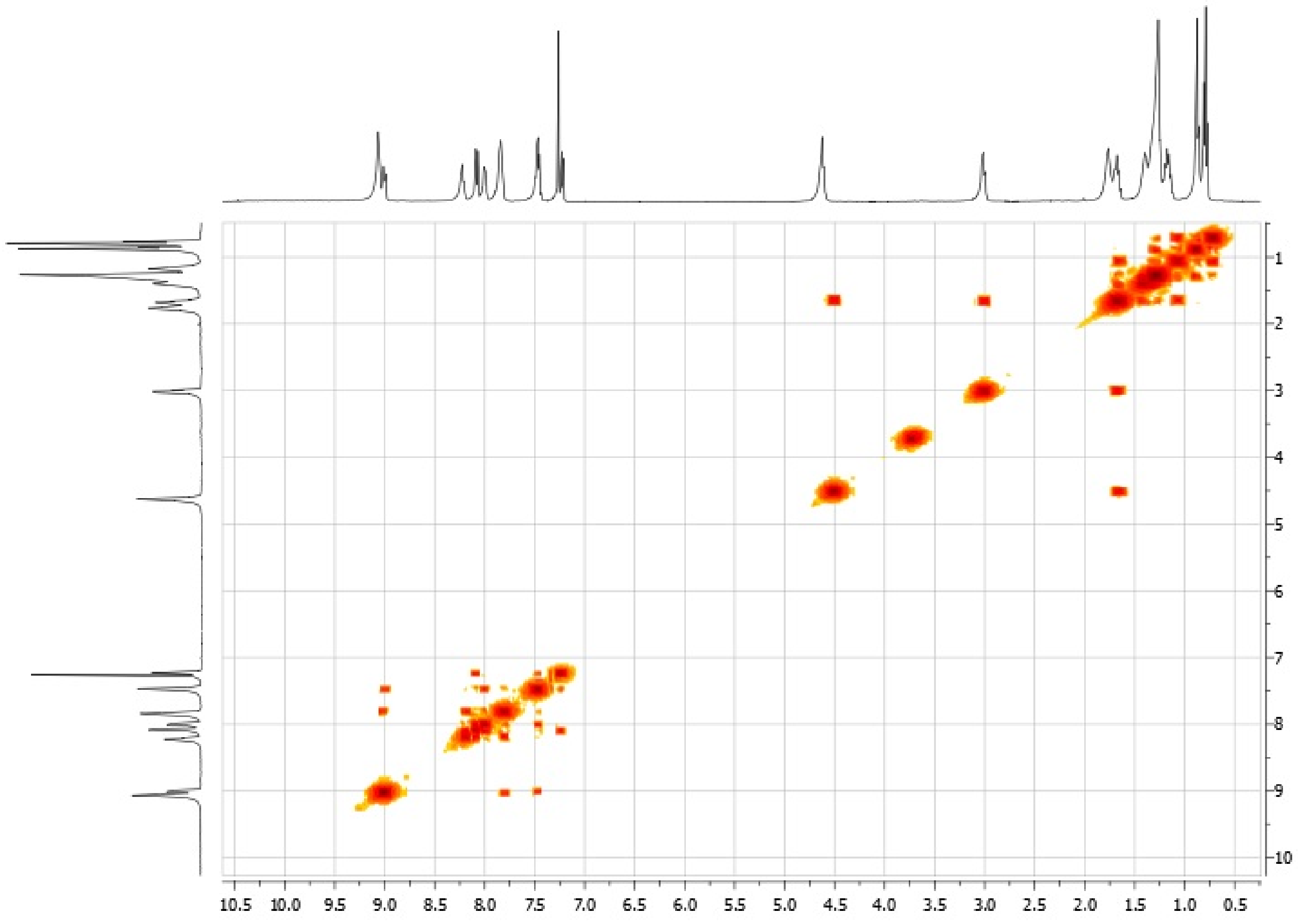

Figure S22. H-H COSY spectrum of [C_4Py][ONS] (**6**)

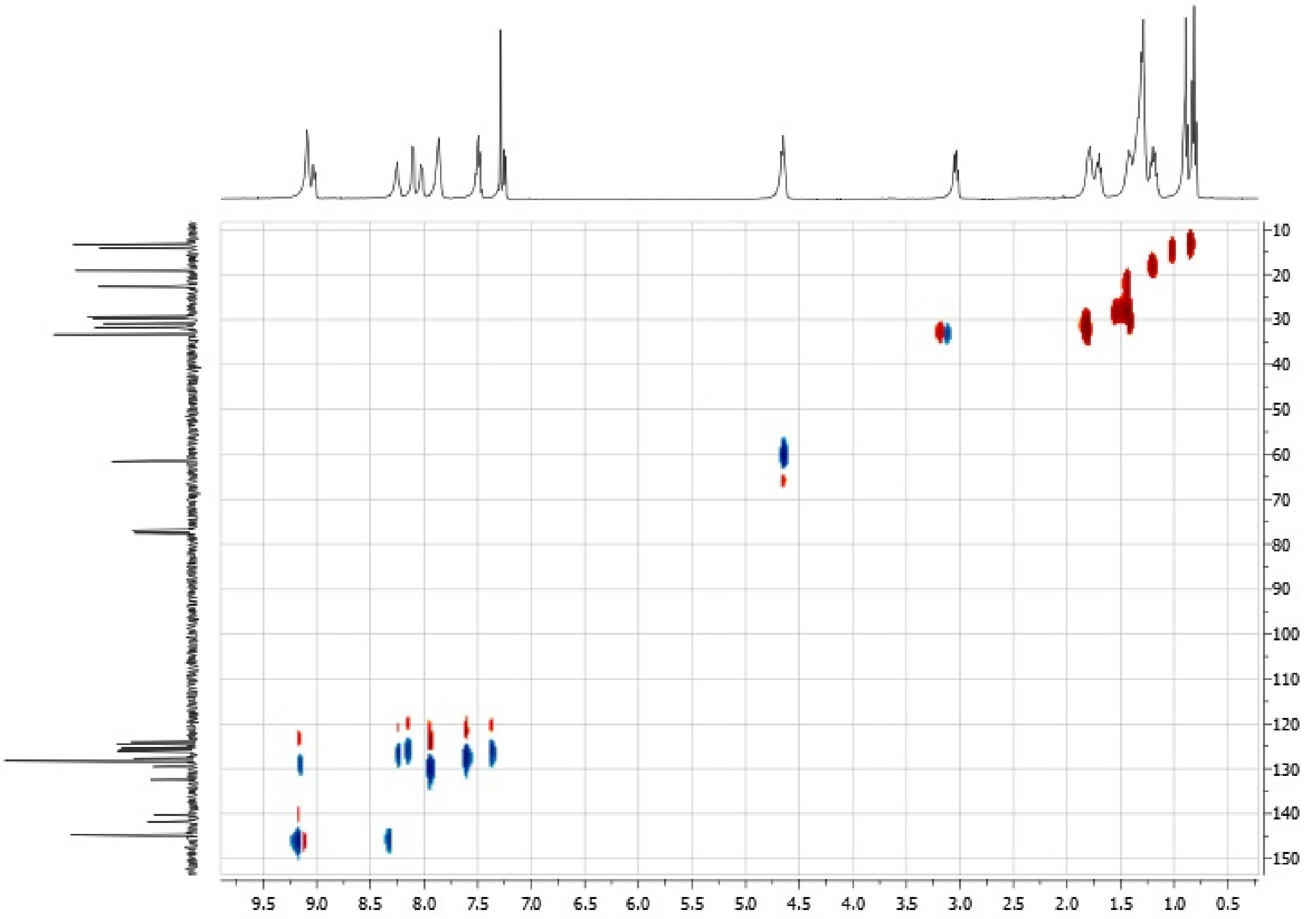

Figure S23. HSQC spectrum of [C_4Py][ONS] (**6**)

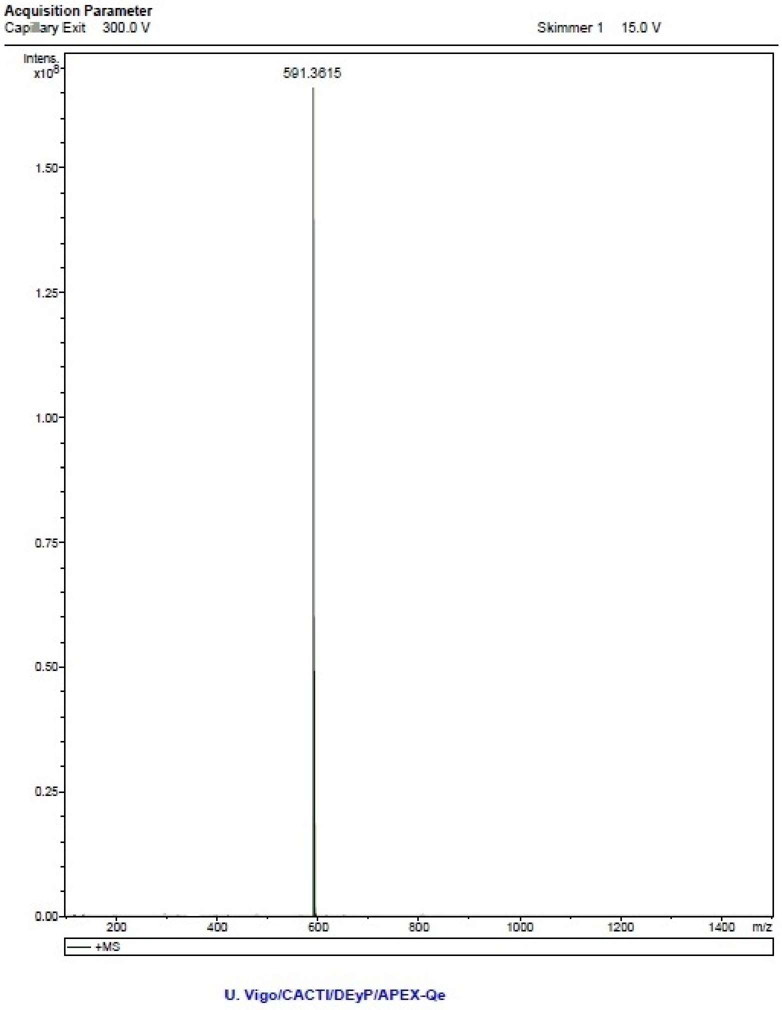

Mass Spectrum Molecular Formula Report

Meas. m/z	#	Ion Formula	m/z	err [ppm]	mSigma	# mSigma	Score	rdb	e⁻ Conf	N-Rule
591.3596	1	C36H51N2O3S	591.3615	3.1	n.a.	3	63.06	12.5	even	ok

Figure S24. HRMS spectrum of [C_4Py][ONS] (**6**)

2.- THERMAL CHARACTERIZATION DETAILS

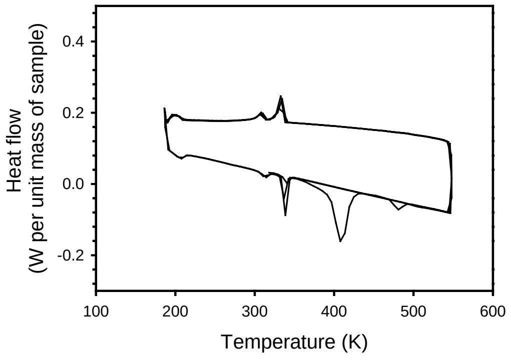

Na[ONS]

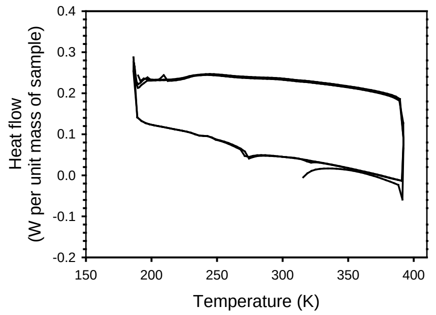

[C_1Pyr][ONS]

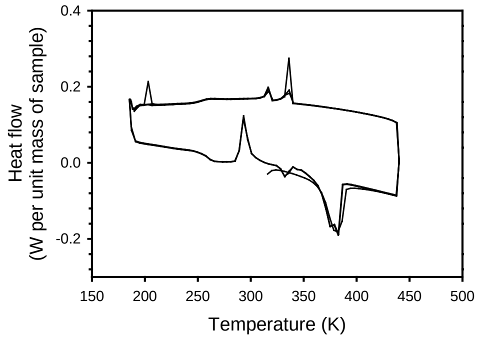

[C_1C_6im][ONS]

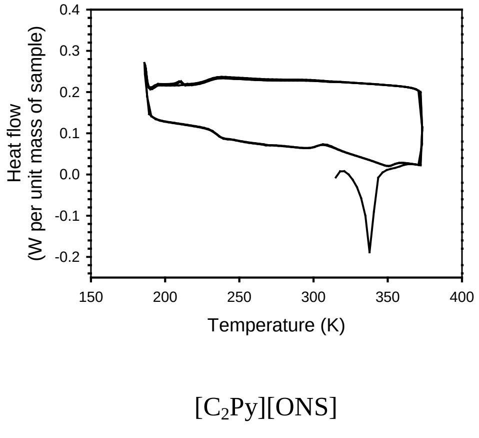

[C_2Py][ONS]

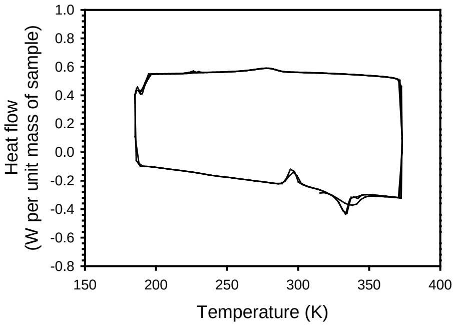

[C_4Py][ONS]

Figure S25. DSC scans carried out at 3 $K \cdot min^{-1}$

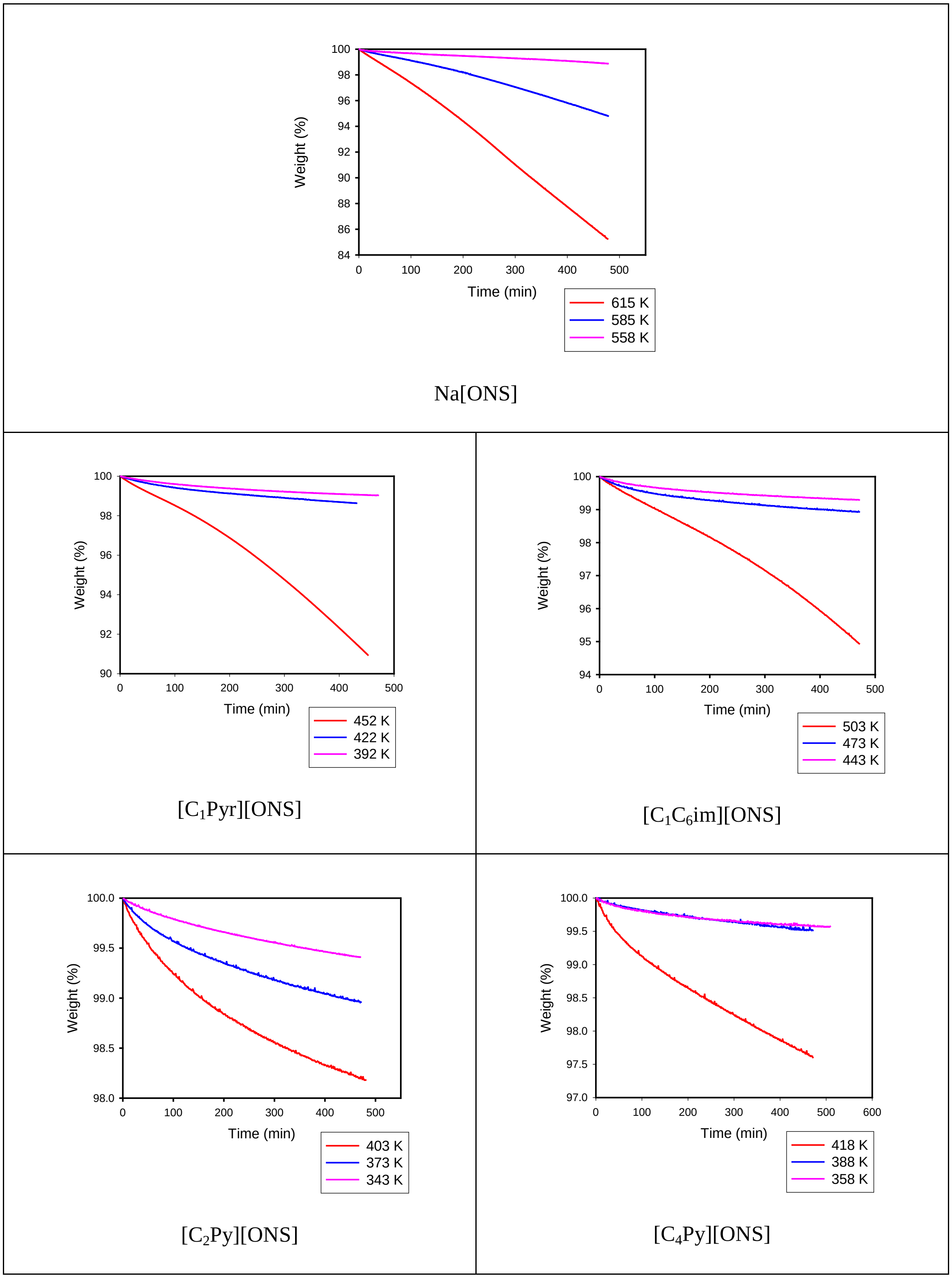

Figure S26. Isothermal TGA curves

References

1. J.P. Nietfeld, R.L. Schwiderski, T.P. Gonella, S.C. Rasmussen, Structural effects on the electronic properties of extended fused-ring thieno[3,4-b]pyrazine analogues, J. Org. Chem. 76 (2011) 6383-6388. DOI:10.10.1021/jo200850w

2. X. Tan, L. Zhang, S. Zhao, J. Yu, J. An, Synthesis and study of the surface properties of long-chain alkylnaphthalene sulfonates, J. Surfactants and Detergents 7 (2004) 135-139. DOI:10.1007/s11743-004-0296-8

3. A. Pal, M. Saini, Aggregation Behavior of Task-Specific Acidic Ionic Liquid N-Methyl-2-Pyrrolidinium Dihydrogen Phosphate [NMP][H_2PO_4] in Aqueous and Aqueous Salt Solutions, J. Surfact. Deterg. 22 (2019) 491-499. DOI:10.1002/jsde.12232
4. G. H. Min, T. Yim, H. Yeong Lee, D. H. Hu, E. Lee, J. Mun, S. M. Oh, Y. G. Kim, Synthesis and Properties of Ionic Liquids: Imidazolium Tetrafluoroborates with Unsaturated Side Chains, Bull. Korean Chem. Soc. 27 (2006) 847-852. DOI: 10.5012/bkcs.2006.27.6.847
5. M. Potangale, A. Das, S. Kapoor, S. Tiwari, Effect of anion and alkyl chain length on the structure and interactions of N-alkyl pyridinium ionic liquids, J. Mol. Liquids (2017) 694–707. DOI: 10.1016/j.molliq.2017.05.036

6. H. Srour, H. Rouault, C. C. Santini, Y. Chauvin, A silver and water free metathesis reaction: a route to ionic liquids, *Green Chem*. 15 (2013) 1341-1347. DOI: 10.1039/C3GC37034H
